# Characterization and anti-inflammatory effect of selenium-enriched probiotic *Bacillus amyloliquefaciens* C-1, a potential postbiotics

**DOI:** 10.1038/s41598-023-40988-8

**Published:** 2023-08-31

**Authors:** Jin Liu, Lu Shi, Xinxin Ma, Sijin Jiang, Xinyao Hou, Pu Li, Yue Cheng, Jia Lv, Shaoru Li, Tianyou Ma, Bei Han

**Affiliations:** 1 School of Public Health, Health Science Center, Xi’an Jiaotong University, Xi’an, 710061 Shaanxi China; 2 Key Laboratory for Disease Prevention and Control and Health Promotion of Shaanxi Province, Xi’an, 710061 Shaanxi China

**Keywords:** Applied microbiology, Industrial microbiology, Biomaterials

## Abstract

A patented strain of *Bacillus amyloliquefaciens* C-1 in our laboratory could produce functional sodium selenite (Na_2_SeO_3_) under optimized fermentation conditions. With the strong stress resistance and abundant secondary metabolites, C-1 showed potential to be developed as selenium-enriched postbiotics. C-1 has the ability to synthesize SeNPs when incubated with 100 μg/ml Na_2_SeO_3_ for 30 h at 30 °C aerobically with 10% seeds-culture. The transformation rate from Na_2_SeO_3_ into SeNPs reached to 55.51%. After selenium enrichment, there were no significant morphology changes in C-1 cells but obvious SeNPs accumulated inside of cells, observed by scanning electron microscope and transmission electron microscope, verified by energy dispersive X-ray spectroscopy and X-ray photoelectron spectroscopy. SeNPs had antioxidant activity in radical scavenge of superoxide (O_2_^−^), Hydroxyl radical (OH^−^) and 1,1-diphenyl-2-picryl-hydrazine (DPPH), where scavenging ability of OH^−^ is the highest. Selenium-enriched C-1 had obvious anti-inflammatory effect in protecting integrity of Caco-2 cell membrane destroyed by *S. typhimurium*; it could preventing inflammatory damage in Caco-2 stressed by 200 μM H_2_O_2_ for 4 h, with significantly reduced expression of IL-8 (1.687 vs. 3.487, *P* = 0.01), IL-1β (1.031 vs. 5.000, *P* < 0.001), TNF-α (2.677 vs. 9.331, *P* < 0.001), increased Claudin-1 (0.971 vs. 0.611, *P* < 0.001) and Occludin (0.750 vs. 0.307, *P* < 0.001). Transcriptome data analysis showed that there were 381 differential genes in the vegetative growth stage and 1674 differential genes in the sporulation stage of C-1 with and without selenium-enrichment. A total of 22 ABC transporter protein-related genes at vegetative stage and 70 ABC transporter protein-related genes at sporulation stage were founded. Genes encoding MsrA, thiol, glutathione and thioredoxin reduction were significantly up-regulated; genes related to ATP synthase such as *atpA* and *atpD* genes showed down-regulated during vegetative stage; the flagellar-related genes (*flgG*, *fliM*, *fliL*, and *fliJ*) showed down-regulated during sporulation stage. The motility, chemotaxis and colonization ability were weakened along with synthesized SeNPs accumulated intracellular at sporulation stage. *B. amyloliquefaciens* C-1 could convert extracellular selenite into intracellular SeNPs through the oxidation–reduction pathway, with strong selenium-enriched metabolism. The SeNPs and selenium-enriched cells had potential to be developed as nano-selenium biomaterials and selenium-enriched postbiotics.

## Introduction

Selenium (Se) is an essential trace element (less than 0.01% of human body weight) for host with important biological functions^1^. Selenium deficiency have an adverse effect on health, such as chronic selenium deficiency adversely affects the function of cardiovascular system and may be a direct cause of heart infarction^2–4^. Selenium deficiency also leads to low immunity^5^, neurological dysfunction^6,7^, and Kashin-beck disease^8^. Selenium is mainly accumulated in human body through food intake. Currently, there are various forms of selenium supplements commercially available, but most of them are inorganic selenium, such as sodium selenite. Since the effective amount of selenium is close to the toxic amount, the inorganic selenium had a narrow safety range, together with the lower biological activity^9^, therefore, finding a new type of selenium supplement (high biological activity and low toxicity) has become research hotspots.

Recently, selenium nanoparticles (SeNPs), a new type of selenium supplement obtained attentions. Studies showed that SeNPs have more physiological functions, such as anti-inflammatory^10^, antibacterial, antioxidant^11^ and anti-tumor^12^. Now, the main methods for SeNPs producing are through chemical and biological processes. The chemical process mainly involves the reduction of Na_2_SeO_3_ to SeNPs by adding active agents in the redox process, which has lower recovery rate and more by-products of pollutants^13^. The biological process is the bio-conversion reaction in bacterial cells, which is efficiency and the whole process is environment friendly, so it is considered as the best choice for SeNPs preparation^14^.

Probiotics, as known of lactic acid bacteria, yeast, *Bifidobacterium* and *Bacillus*, are beneficial to host health in improving intestinal environment, preventing diseases by lowering intestinal pH and maintaining gut microbiota^15^. In addition, probiotics also have functions of resisting pathogens infestation and regulating host immunity^16^. Postbiotics are a new category of biotics that have the potential to confer health benefits, but unlike probiotics, do not require living cells. Postbiotics are defined as a "preparation of inanimate microorganisms and/or their components that confers a health benefit on the host" by the consensus panel of the International Scientific Association of Probiotics and Prebiotics (ISAPP). Postbiotic components include short-chain fatty acids, exopolysaccharides, vitamins, teichoic acids, bacteriocins, enzymes and peptides in a non-purified inactivated cell preparation. Postbiotics have technological advantages over probiotics incorporated into foods, since they are more stable, and their storage, handling, and transportation can be more economically feasible^17^.

It is reported that some probiotics can metabolize selenium in growth medium and convert Na_2_SeO_3_ into red SeNPs through cell biotransformation^18,19^. *Bacillus* is gram-positive aerobic bacterium that has been used as a probiotic for at least 60 years. It reported that *B. megaterium* ATCC 55,000 can reduce Na_2_SeO_3_ to SeNPs^20^. Akçay et al. isolated a strain of *B. megaterium* EKT1 from soil and demonstrated its ability to biosynthesize SeNPs^21^. Therefore, if the performance of SeNPs prepared by reduction of *Bacillus* probiotic is combined with its probiotic characters, the complex of *Bacillus* and SeNPs may be developed as postbiotics, which can not only provide enough selenium, and improve intestinal health. Foods with bacterial probiotics and postbiotics are premised on being healthier than those not incorporated with them. Therefore, developing food products with bacterial probiotics and postbiotics is a great opportunity for research in food science, medicine and nutrition, as well as in food industry^17^.

To obtain highly efficiency anti-inflammatory, antioxidant, safe and non-toxic composite nano-selenium materials and prepare for the postbiotics development, *B. amyloliquefaciens* C-1, a patent strain isolated and stored in our laboratory, was used to biosynthesize SeNPs. The Se-enriched culture conditions were optimized, the produced SeNPs was characterized, and the biofunctions and safety were evaluated.

## Results

### Optimization of selenium-enriched culture condition for B*. amyloliquefaciens* C-1

**Figure 1:**
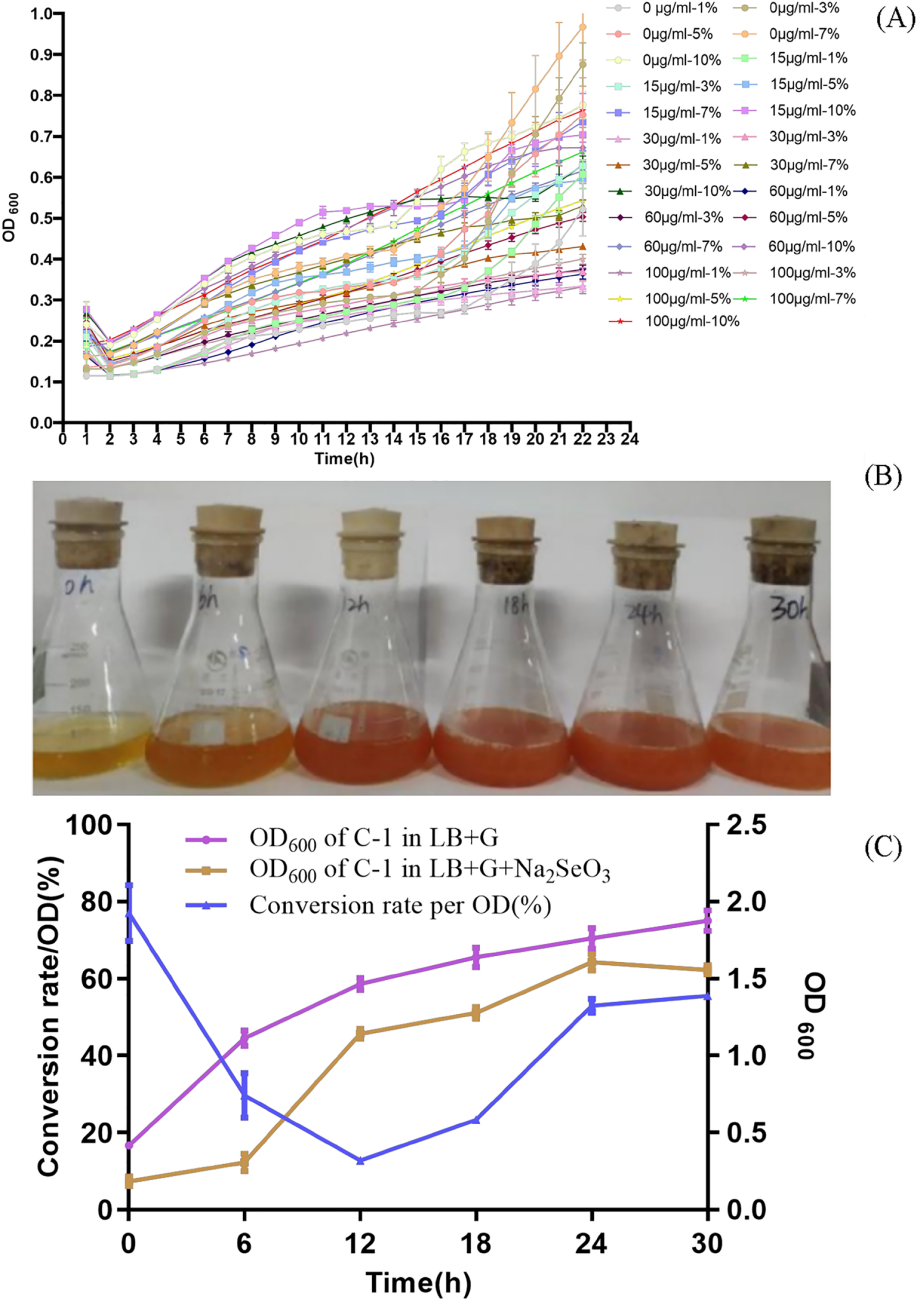
Synthesis of SeNPs and preparation of selenium-enriched *B. amyloliquefaciens* C-1. The 1, 3, 5, 7, 10% fresh overnight culture of C-1 were sub-cultured in LB + G medium with 0, 15, 30, 60, 100, 150 μg/ml selenite, separately. And the growth of C-1 was monitored by measuring OD_600_ every 60 min at 30 °C for 22 h (**A**); the fermentation culture of C-1 in LB + G medium with 100 μg/ml Na_2_SeO_3_ at 0, 6, 12, 18, 24 and 30 h (**B**); selenium conversion rate of C-1 in LB + G medium with 100 μg/ml Na_2_SeO_3_ and with 10% inoculum, the cells were collected every 6 h. Selenium were determined by spectrophotometric method (**C**).

The growth curves of C-1 at fermentation medium supplied with 0–150 μg/ml of Na_2_SeO_3_ are shown in Fig. 1A. It showed that C-1 could grow well at 100 μg/ml of Na_2_SeO_3_ and a 10% seeds-culture inoculum. To confirm the optimal fermentation time for selenium-enriched C-1 cells, C-1 was continuously incubated with 100 μg/ml of Na_2_SeO_3_ and a 10% inoculum for 30 h at 30 °C. The OD_600_ was measured and results are shown in Fig. 1B.With an increased incubation time the red color of C-1 cells got stronger, which indicated the production of SeNPS (Fig. 1C). It showed that *B. amyloliquefaciens* C-1 had a maximum Se conversion rate of 55.51% per OD at 30 °C at 30 h fermentation.

### Characterization of selenium-enriched C-1 cells and SeNPs

**Figure 2:**
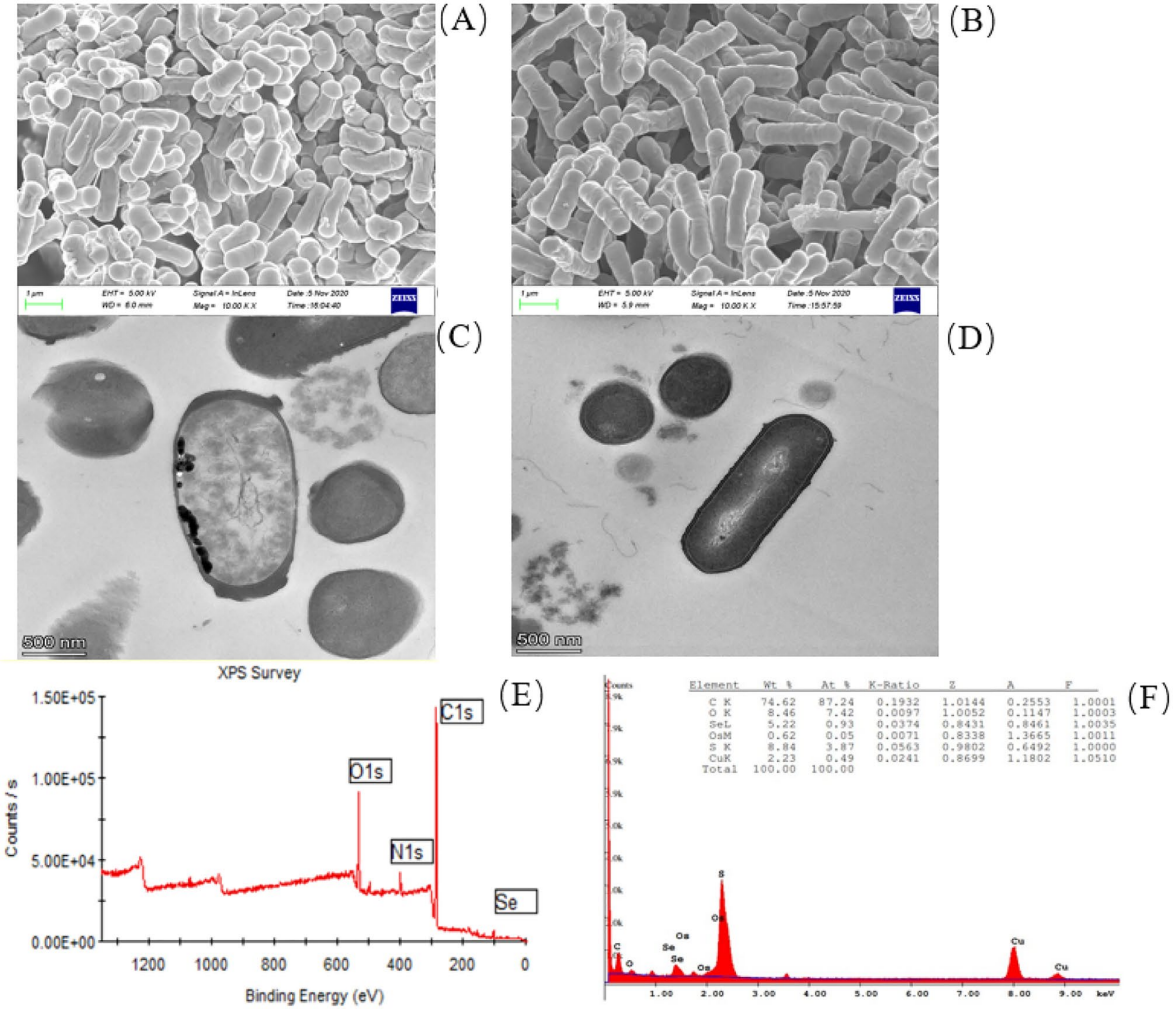
Characterization of selenium-enriched *B. amyloliquefaciens* C-1 and the produced SeNPs. The C-1 cells morphology was observed by SEM cultivated with (**A**) and without (**B**) 100 μg/ml Na_2_SeO_3_, by TEM cultivated with (**C**) and without (**D**) 100 μg/ml Na_2_SeO_3_; and the composition of carbon, nitrogen, oxygen, phosphorus, sulfur and selenium elements in Se-enriched C-1 were analyzed by XPS (**E**) and EDS (**F**).

The SEM and TEM analysis showed that there was no significant difference in morphology between selenium-enriched *B. amyloliquefaciens* C-1 cells (Fig. 2A, C) and C-1 cells (Fig. 2B, D), and the vegetative cells were all in rod shape. TEM observations revealed that the biosynthesized SeNPs produced by C-1 were deposited inside of the cell and released to the extracellular after cell lysis. The energy spectra of the SeNPs were observed by XPS, and the results showed that the carbon (C), nitrogen (N), oxygen (O) and selenium (Se) were all detected in the cell biomass(Fig. 2E).The results of EDX analysis showed in Fig. 2F and indicated the presence of carbon (C), oxygen (O), selenium (Se), osmium (Os), sulfur (S) and copper (Cu) with relative concentration of 74.62, 8.46, 5.22, 0.62, 8.84 and 2.23%, respectively within selenium-enriched C-1 cells.

### Free radical scavenging activity

**Figure 3:**
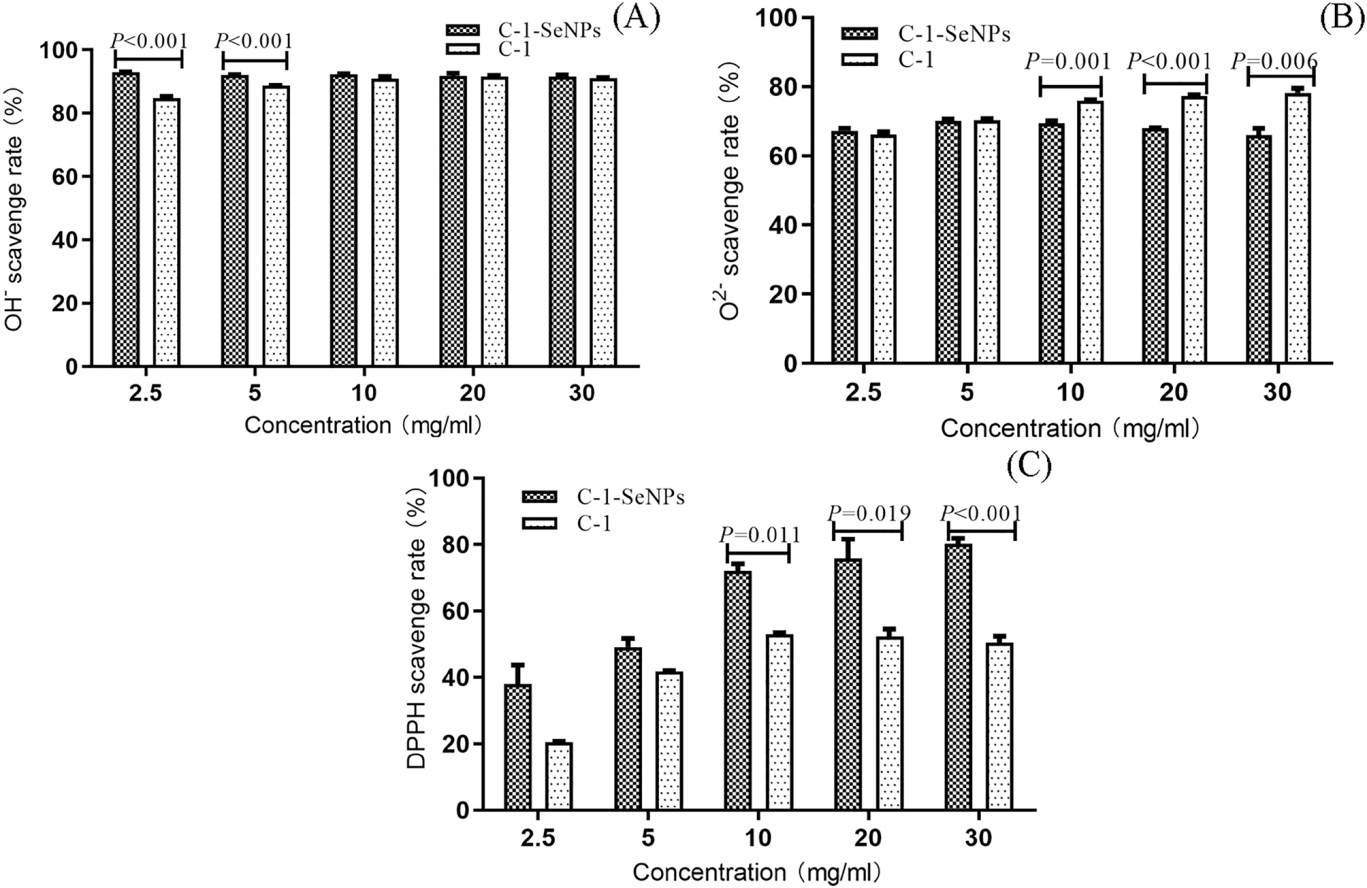
Antioxidant properties of selenium-enriched C-1 in scavenging rate of superoxide radical (**A**), hydroxyl radical (**B**), DPPH radical (**C**). The statistical significance was calculated by one-way ANOVA.

The free radical scavenging ability of selenium-enriched C-1 and C-1 on O_2_^−^, OH^-^and DPPH were investigated, and the results are shown in Fig. 3.

Figure 3A showed that both selenium-enriched C-1 and C-1 had the ability to scavenge O_2_^−^. And C-1 exhibited a higher scavenging ability than selenium-enriched C-1 when the concentration was higher than 10 mg/ml (10 mg/ml: 75.95% vs. 69.47%, *P* = 0.001; 20 mg/ml: 77.42% vs. 67.93%, *P* < 0.001; 30 mg/ml: 78.24% vs. 65.99%, *P* = 0.006). Figure 3B showed that both selenium-enriched C-1 and C-1 had the ability to scavenge OH^-^. And selenium-enriched C-1 exhibited a higher scavenging ability than C-1 when lower than 5 mg/ml (2.5 mg/ml: 92.90% vs. 84.81%, *P* < 0.001; 5 mg/ml: 92.10% vs. 88.75%, *P* < 0.001). And there was no significant difference in the scavenging ability of different concentrations of selenium-enriched C-1, which were more than 92%. Figure 3C showed that selenium-enriched C-1 exhibited a higher scavenging ability than C-1 for DPPH radicals scavenging, with a dose-dependent manner, especially in more than 10 mg/ml (10 mg/ml: 71.98% vs. 52.95%, *P* = 0.001; 20 mg/ml: 75.85% vs. 52.31%, *P* = 0.019; 30 mg/ml: 80.16% vs. 50.42%, *P* < 0.001).

### Protect effect to Caco-2 cell

#### Cytotoxicity on Caco-2 cell

For cell viability assay treated with different concentrations of C-1 and selenium-enriched C-1 bacterial cells, compared with the untreated control group, both the C-1 and selenium-enriched treatment had no toxicity on the growth of Caco-2 cells at concentrations of 8 × 10^3^, 1 × 10^4^, 8 × 10^4^, 1 × 10^5^ CFU/ml (*P* > 0.05) (Table 1). In the following experiments, the concentrations of 8 × 10^3^, 1 × 10^4^, 8 × 10^4^ and 1 × 10^5^ CFU/ml of C-1 and selenium-enriched C-1 were selected when treated the Caco-2 cells.

**Table 1:** The relative cell viability of C-1 and selenium-enriched C-1 to Caco-2 cell measured by MTT.

Concentration (CFU/ml)	C-1		Selenium-enriched C-1		*t*	*P*
	Relative cell viability (%)	STD (%)	Relative cell viability (%)	STD		
8 × 10^3^	94.83	4.32	100.10	3.34%	− 2.115	0.063
1 × 10^4^	93.43	3.26	92.54	4.96%	0.309	0.770
8 × 10^4^	93.99	5.84	100.00	6.55%	− 1.456	0.189
1 × 10^5^	94.74	2.60	92.71	4.18%	0.848	0.437
8 × 10^5^	100.74	10.33	71.82	6.07%	5.757	< 0.001
1 × 10^6^	96.81	10.83	77.82	6.68%	3.575	0.006
8 × 10^6^	96.78	3.88	56.81	3.70%	17.426	< 0.001
Control	100		100			

#### Protect effect on Caco-2 cell membrane integrity stressed by *S. typhimurium*

**Figure 4:**
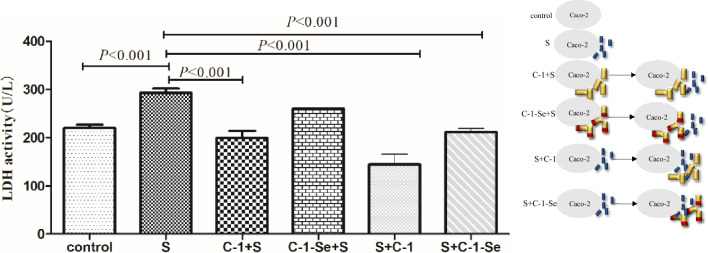
Protect effect of selenium-enriched *B. amyloliquefaciens* C-1 to Caco-2 cell membrane integrity challenged by *S. typhimurium* ATCC14028 by LDH activity assay. S (*S. typhimurium* ATCC14028 treatment); C-1+S (2 h incubation with C-1 firstly, then *S. typhimurium* added and incubated for another 2 h); S+C-1 (2 h incubation with *S. typhimurium* firstly, then C-1 added and incubated for another 2 h); C-1-Se+S (2 h incubation with selenium-enriched C-1 firstly, then *S. typhimurium* added and incubated for another 2 h); S-C-1+Se (2 h incubation with *S. typhimurium* firstly, then selenium-enriched C-1 added and incubated for another 2 h). The statistical significance was calculated by one-way ANOVA.

The cell membrane integrity of Caco-2 cells was evaluated by detecting LDH activity in cell supernatant, and results are shown in Fig. 4. Compared with control group, the treatment with *S. typhimurium* ATCC14028 significantly increased LDH activity (292.99 U/L vs. 219.94U/L, *P* < 0.001), which indicated the induce of cell damages. The S+C-1 and S+C-1-Se groups had significantly lower LDH activities than *S. typhimurium* damage group (144.58U/L vs. 219.94 U/L, *P* < 0.001; C-1-Se: 111.35 U/L vs. 219.94 ± 11.94 U/L, *P* < 0.001), which indicated the damage to cell membrane caused by *S. typhimurium* may be partially repaired by probiotics.

#### Protect effect on oxidative stressed Caco-2 induced by H_2_O_2_

The viability of Caco-2 cells gradually decreased with the increase concentration of H_2_O_2_, proving that the cells were suffered to oxidative stress. Compared with the negative control (100% cell viability), the cell viability decreased into 98.27, 89.41, 57.47, 47.41,47.13, 44.33 and 42.49% separately after 4 h treatment by 100, 200, 400, 800, 1200, 1600 and 2000 μM H_2_O_2_ (*P* < 0.001). For the following experiment, the 400 μM H_2_O_2_ was used to construct the cell stress model.

**Figure 5:**
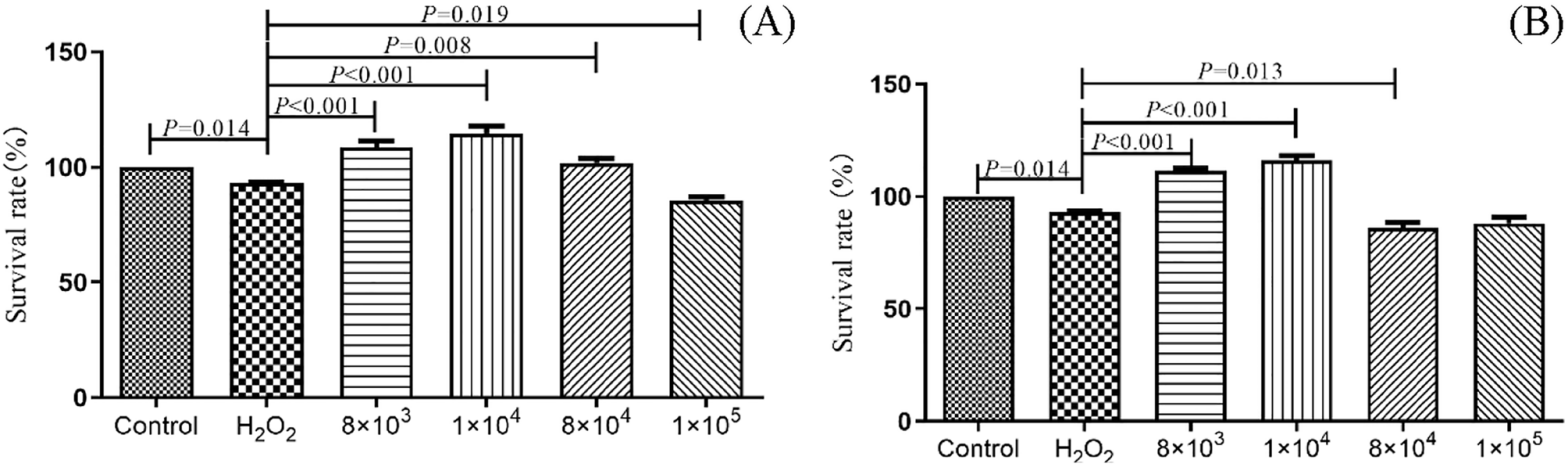
The effect of *B. amyloliquefaciens* C-1 (**A**) and Se-enriched C-1 (**B**) on oxidative stressed Caco-2 cells induced by H_2_O_2_, showed in cell viability. The statistical significance was calculated by one-way ANOVA.

As shown in Fig. 5, compared with the H_2_O_2_-stressed group, the pretreat of C-1 and selenium-enriched C-1 groups can alleviate the H_2_O_2_-induced cell growth inhibition. Under incubation of 8 × 10^3^ and 1 × 10^4^ CFU/ml bacteria cells, the effect was most significant (C-1:108.51% vs. 60.12%, *P* < 0.001; 114.5% vs. 60.12%, *P* < 0.001; Selenium-enriched C-1: 111.53% vs. 60.12%, *P* < 0.001; 115.88% vs. 60.12%, *P* < 0.001). And selenium-enriched C-1 and C-1 of 8 × 10^3^ and 1 × 10^4^ CFU/ml were chosen as pre-treatment in following tests to observe the inflammatory cytokines expression.

#### Effect on inflammatory cytokines expression in oxidatively stressed Caco-2

**Figure 6:**
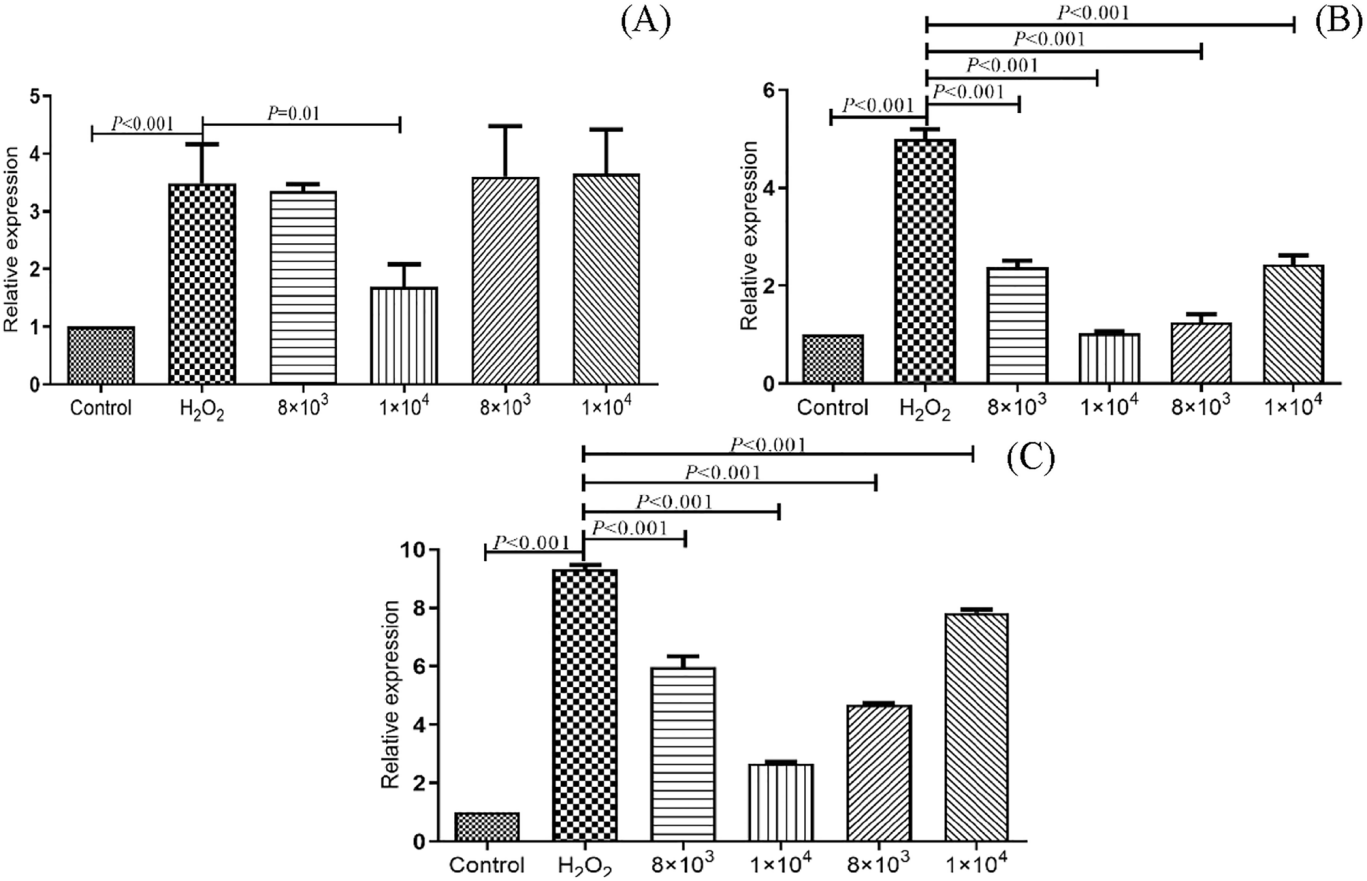
Effects of Se-enriched *B. amyloliquefaciens* C-1 on inflammatory factors of IL-8 (**A**), IL-1β (**B**) andTNF-α (**C**) in oxidatively stressed Caco-2 cells. The statistical significance was calculated by one-way ANOVA.

q-RT-PCR was used to detect the mRNA relative expression levels of cytokines IL-8, IL-1β and TNF-α, and the results were shown in Fig. 6A-C. Compared with negative control, mRNA expression levels of IL-8, IL-1β and TNF-α in oxidative model were significantly increased for 3.487 times, 5.000 times, 9.331 times, separately (*P* < 0.001).

Compared with 3.487 times higher expressed IL-8 in oxidative model, high concentration selenium-enriched C-1 group could significantly reduce it to 1.687 time (*P* = 0.01); Compared with 5.000 times higher expressed IL-1β in oxidative model expression, four treatment groups were all showed a significantly reduced expression of IL-1β (2.432 times in 1 × 10^4^ CFU/ml C-1; 1.258 times in low concentration C-1; 1.031 times in 1 × 10^4^ CFU/ml Selenium-enriched C-1; 2.371 times in 8 × 10^3^ CFU/ml Selenium-enriched C-1; *P* < 0.001). And 1 × 10^4^ CFU/ml Selenium-enriched C-1 treatment had the lowest mRNA expression level of IL-1β.

Compared with 9.331 times higher expressed TNF-α in oxidative model, four treatment groups were all showed a significantly reduced expression of TNF-α (7.837 times in 1 × 10^4^ CFU/ml C-1; 4.687 times in 8 × 10^3^ CFU/ml C-1; 2.677 times in 1 × 10^4^ CFU/ml Selenium-enriched C-1; 2.974 times in 8 × 10^3^ CFU/ml selenium-enriched C-1; *P* < 0.001). And the mRNA expression level of TNF-α was lowest after treatment with 1 × 10^4^ CFU/ml Selenium-enriched C-1. All the results showed the high ability of selenium-enriched C-1 to relieve oxidative stress of Caco-2 cells.

#### Protect effect on the tight junction state of Caco-2 under oxidative stress

**Figure 7:**
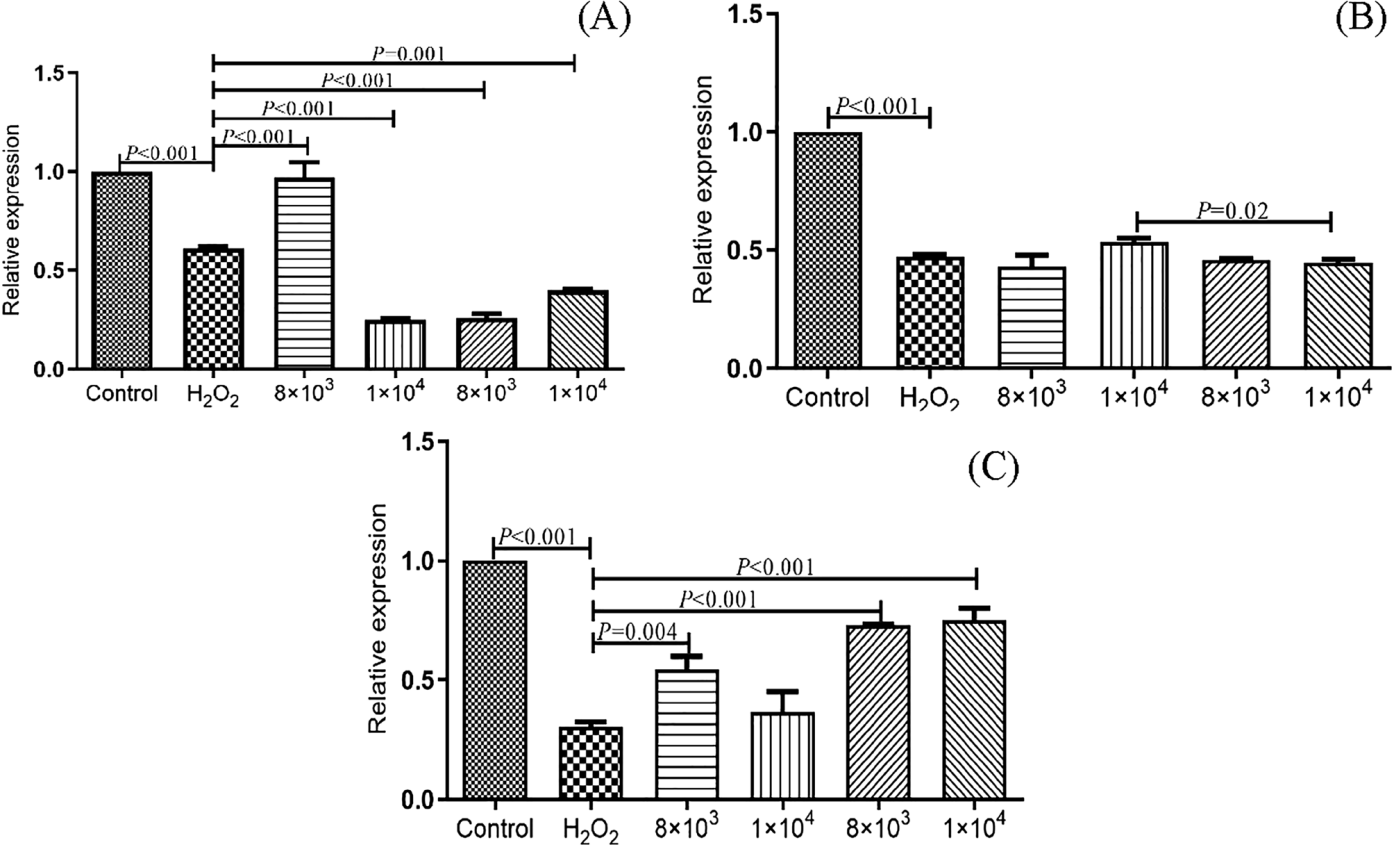
Effects of Se-enriched *B. amyloliquefaciens* C-1 on membrane permeability related proteins of Claudin-1 (**A**), ZO-1 (**B**) and Occludin (**C**) in oxidatively stressed Caco-2 cells. The statistical significance was calculated by one-way ANOVA.

Gene expression levels of cell tight junction structure protein (Claudin-1, ZO-1, Occludin) results are shown in Fig. 7A-C. Compared with control group, the relative expression of Claudin-1, ZO-1 and Occludin in oxidative model (H_2_O_2_ treated group) were significantly decreased into 0.611, 0.472 and 0.307 (*P* < 0.001), which indicated the oxidative stressed of cells reduced cell junction and increased permeability of cell membrane. While the incubation of selenium-enriched C-1 and C-1 significantly restored the tight junction state of cells, then reduced the permeability of cell membrane. Compared with oxidative model, 8 × 10^3^ CFU/ml selenium-enriched C-1 significantly increased the expression level of Claudin-1 (0.971 vs. 0.611, *P* < 0.001); And the expression of Occludin in 1 × 10^4^ CFU/ml C-1 treatment was the highest (0.750 vs. 0.307, *P* < 0.001); the expression of ZO-1 had no significantly increased in the treatment groups (*P* > 0.05).

### Transcriptomic analysis of selenium-enriched metabolism of C-1 cells

RNA was extracted from *B. amyloliquefaciens* C-1 and selenium-enriched C-1 cell. The RNA samples were tested for quality control, and quality trimmed reads were mapped to C-1 using Bowtie2 v2.2.8, reads mapped to ribosome RNA were removed. Retained reads were aligned with reference genome of C-1 to identify known genes and calculated gene expression by RSEM, and the result was summarized in Supplementary Table S2.

**Figure 8:**
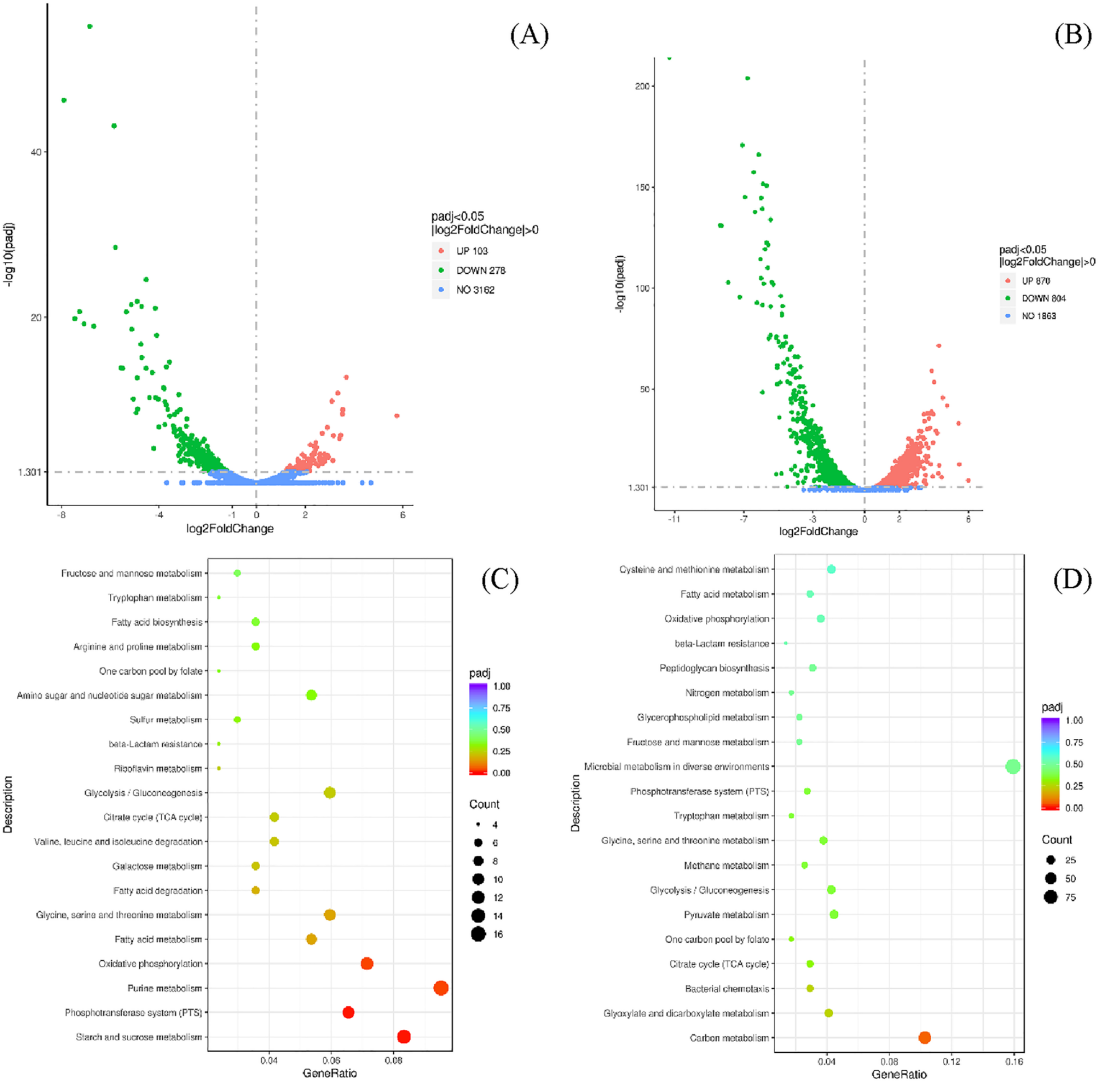
Comparison of gene expression at the transcription level in *B. amyloliquefaciens* C-1 in LB + G medium supplied with and without 100 μg/ml Na_2_SeO_3_. In the Volcano Plot, genes with significantly differential expression (fold change > 0 and *P*_*adj*_ < 0.05; Se-enriched C-1 vs. C-1) were shown in green (down-regulated), red (up-regulated) and blue (non-significantly differential ex-pression) at vegetative stage (**A**) and sporulation stage (**B**). The differently expressed genes were analyzed and shown in KEGG dotplot at vegetative stage (**C**) and sporulation stage (**D**).

In the transcriptome analysis results, there were 381 differential genes at vegetative stage, including 103 up-regulated and 278 down-regulated genes; and there were 1381 differential genes at sporulation stage, including 719 up-regulated and 662 down-regulated genes (DESeq2 with |log2 (Fold Change)|> 1 and *P*_adj_ < 0.05) (Fig. 8A, B, Supplementary Table S3). It indicates that Na_2_SeO_3_ has effect on gene transcription level of C-1, especially at sporulation stage. The differential transcription genes of selenium-enriched C-1 cell compared with C-1 are listed in Table 2 (|log2(Fold Change)|> 5, *P*_adj_ < 0.05).

**Table 2:** Differentially expressed genes in Se-enriched *B. amyloliquefaciens* C-1 versus C-1. (|log2FoldChange|> 5, *P*_*adj*_ < 0.001).

Stage	Gene ID	Description	log2FC
Vegetative stage	RS04830	Alpha/beta-type small acid-soluble spore protein	+ 5.7591
	RS15570	Acyl-CoA dehydrogenase family	− 7.9031
	RS07680	S8 family serine peptidase	− 7.4537
	RS03985	Dihydrolipoyl dehydrogenase	− 7.2566
	RS03980	2-oxo acid dehydrogenase subunit E2	− 7.0734
	RS15575	Acetyl-CoA C-acetyltransferase	− 6.8428
	RS03975	Transketolase, pyrimidine binding domain	− 6.6687
	RS13215	Electron transfer flavoprotein domain	− 5.8396
	RS15580	Enoyl-CoA hydratase/isomerase	− 5.7843
	RS03970	Dehydrogenase E1 component	− 5.5615
	RS09735	Glucosylceramidase	− 5.4867
	RS01920	Amino acid ABC transporter substrate-binding protein	− 5.3382
	RS01930	ABC transporter	− 5.1348
	RS09175	3-hydroxybutyrate dehydrogenase	− 5.1124
	RS01925	Amino acid ABC transporter permease	− 5.0475
Sporulation stage	RS03050	tRNA-Asp	+ 5.9988
	RS13765	Cation transporter	+ 5.4799
	RS04005	MurR/RpiR family transcriptional regulator	+ 5.4426
	RS01675	Hypothetical protein	− 11.3040
	RS03985	Dihydrolipoyl dehydrogenase	− 8.3691
	RS03980	2-oxo acid dehydrogenase subunit E2	− 8.2980
	RS03975	Transketolase, C-terminal domain	− 7.8965
	RS03970	Dehydrogenase E1 component	− 7.2244
	RS07680	S8 family serine peptidase	− 7.0812
	RS15570	Acyl-CoA dehydrogenase family protein	− 6.9295
	RS09155	Non-ribosomal peptide synthetase	− 6.7826
	RS14995	Bacteriocin class IId cyclical uberolysin-like	− 6.4219
	RS05110	S8 family serine peptidase	− 6.3485
	RS09175	3-hydroxybutyrate dehydrogenase	− 6.2301
	RS11395	SDR family NADP-dependent oxidoreductase	− 6.1285
	RS08890	Pectate lyase superfamily protein	− 6.0259
	RS09185	Coenzyme A transferase	− 5.9925
	RS15575	Acetyl-CoA C-acetyltransferase	− 5.9870
	RS09160	Non-ribosomal peptide synthetase	− 5.9227
	RS09180	CoA transferase subunit B	− 5.9106
	RS04195	Hypothetical protein	− 5.9089
	RS07505	Cytochrome c oxidase assembly factor CtaG	− 5.8931
	RS08870	Lytic polysaccharide monooxygenase	− 5.8423
	RS11390	Ketoacyl-synthetase C-terminal	− 5.7588
	RS07500	Cytochrome c oxidase subunit IVB	− 5.6769
	RS09165	Non-ribosomal peptide synthetase	− 5.6738
	RS11400	Polyketide synthase dehydratase	− 5.6150
	RS07495	Cytochrome c oxidase subunit III	− 5.5882
	RS15935	Zinc-dependent metalloprotease	− 5.5505
	RS02915	Methyltransferase domain	− 5.4510
	RS07490	Cytochrome c oxidase subunit I	− 5.4474
	RS11385	Zinc-binding dehydrogenase	− 5.4464
	RS11405	SDR family NAD(P)-dependent oxidoreductase	− 5.3735
	RS11410	KR domain-containing protein	− 5.2908
	RS06245	S9 family peptidase	− 5.1361
	RS06250	PhrA family phosphatase inhibitor	− 5.1220
	RS09190	GntP family permease	− 5.1065
	RS10215	Hypothetical protein	− 5.0673
	RS02910	NUDIX hydrolase	− 5.0636
	RS11380	Cytochrome P450	− 5.0568

Enrichment analysis of those different transcript genes was performed to explore the biological effects and the corresponding pathways that are significantly associated in GO and KEGG analysis. In GO functional enrichment analysis (Supplementary Table S4), all differential genes were significantly enriched into 3 GO Terms, including protein-containing complex-hydrolase activity, hydrolyzing O-glycosyl compounds, hydrolase activity-acting on glycosyl bonds at vegetative stage. At sporulation stage, all differential genes were significantly enriched into 42 GO Terms and they are all belonging to Biological Process. There were 3 terms that were down-regulated, including chemotaxis, taxis and locomotion. It suggested that the chemotaxis and locomotion of *B. amyloliquefaciens* decreased when Na_2_SeO_3_ supplied in the fermentation medium. There were 39 terms that were up-regulated, the terms in which the most different-enriched genes were mainly involved in nucleobase-containing compound metabolic process, nucleic acid metabolic process, cellular aromatic compound metabolic process, heterocycle metabolic process, cellular nitrogen compound metabolic process, organic cyclic compound metabolic process, cellular biosynthetic process, organic substance biosynthetic process, macromolecule metabolic process, etc.

In the KEGG pathways enrichment analysis (Supplementary Table S5), all differential genes were significantly enriched into 4 pathways (starch and sucrose metabolism, phosphotransferase system, purine metabolism, oxidative phosphorylation) at vegetative stage, which were all up-regulated (Fig. 8C). At sporulation stage, all differential genes were significantly enriched into 8 pathways (Fig. 8D), where there were 2 up-regulated pathways (glycerophospholipid metabolism, fructose and mannose metabolism), and 6 down-regulated pathways (citrate cycle, bacterial chemotaxis, glycolysis/gluconeogenesis, glyoxylate and dicarboxylate metabolism, oxidative phosphorylation and carbon metabolism). This indicated that the metabolism activity of C-1 with addition of Na_2_SeO_3_ decreased in the later growth stage, the accumulation of metabolic waste would inhibit the growth, and its chemotaxis and colonization capacity decreased, which it is consistent with the bio-conversion curve of Na_2_SeO_3_ to SeNPs by C-1 (Fig. 1B).

The q-RT-PCR results of three randomly selected differentially expressed genes are shown in Supplementary Fig. S1. The expression of these three genes were consistent with the results of transcriptome analysis with R^2^ of 0.966.

## Discussion

Selenium is an essential biological element for all living organisms. The amount of selenium in human tissues varies by diet. Chronic deficiency of selenium could lead to serious diseases^2–4^. In recent years, with the increasing of dietary supplement of selenium, the application of microbial reduction of selenite into SeNPs has gained attention. However, there exists limitations in using microorganisms to synthesize SeNPs, such as strict anaerobic environment for lactic acid bacteria growth, expensive fermentation equipment and higher costs^22^. While the development of selenium-enriched probiotics with aerobic bacteria maybe another better choice.

*Bacillus* probiotics are aerobically reproduced and could regulate the gut microbiota, improve host immunity and reduce the intestinal diseases by colonization in the intestinal tract. Ashengroph et al. isolated a strain of *Bacillus* SRB04 from coast of Caspian Sea and confirmed its selenium-enriching ability^23^. Fischer et al. isolated *Bacillus safensis* JG-B5T from soil and demonstrated that it produced unstable SeNPs^24^. Similarly, in this study, we found that in concentration of 100 μg/ml Na_2_SeO_3_, 10% C-1 seed culture, incubated at 30 °C for 30 h, *B. amyloliquefaciens* C-1 obviously reduced Na_2_SeO_3_ to SeNPs with a high conversion rate of 55.51% per OD.

It was reported that the number of bacteria cells decreased after selenium-enriched fermentation of *B. paralicheniformis* SR14 without any morphological change^25^. In our study, the addition of Na_2_SeO_3_ in medium also led to reducing of C-1 cell number, indicating the toxicity of inorganic Na_2_SeO_3_ to bacteria cells. While, there had no morphology changes for C-1 cells by SEM. The synthesis site of SeNPs can be intracellular, extracellular or membrane-bound. Bacteria-produced SeNPs accumulated intracellularly in the middle and late stages of the exponential growth period, and were secreted into the medium during the stationary phase. Liu et al. isolated a probiotic *Enterococcus durans* A8-1 and confirmed the accumulated SeNPs intracellular and extracellular^26^. The TEM results of this study showed that the SeNPs were obviously aggregated inside of C-1 cells.

For the observed cytotoxicity of selenium-enriched C-1 at concentrations of more than 8 × 10^5^ CFU/ml, Studies have shown that the toxicity range of SeNPs is wider than that of selenium, but it does not mean that nano-selenium is completely non-toxic, only that it is safe within a certain range^19^. So the safety evaluation of SeNPs, even produced by probiotics is still necessary.

The development of natural and safe antioxidants has attracted attention. SeNPs has been shown with better antioxidant activity, which will become a new supplement to antioxidants. Studies have shown that more than 95% of free radicals belong to oxygen radicals, such as O_2_^−^ and OH^−^ radicals. Unlike oxygen radicals, DPPH are synthetic organic radicals that are widely used to evaluate the antioxidant capacity of biological samples. Shakibaie et al. isolated a strain of selenium-tolerant lactic acid bacteria from traditional Iranian dairy products that could tolerate 3.16 mmol/L selenite, and showed good free radicals scavenging ability^27^. In this study, selenium-enriched C-1 showed scavenging ability against O_2_^−^, OH^-^, and DPPH, where scavenging ability of OH^-^ was the highest.

Caco-2 cells are used to construct models of human intestinal oxidative stress to screen antioxidant in treating and preventing intestinal oxidative damage. Xu et al. found that SeNPs produced by *Lactobacillus casei* ATCC393 could protect intestinal barrier function damage caused by *E. coli* K88, and reduce expression of IL-1β and TNF-α^28^. In our study, the oxidative stress model in Caco-2 cells were induced by H_2_O_2_, and the tested selenium-enriched C-1 had the potential to inhibit the inflammatory response at a certain concentration, and the similar cytokines expression profiles were also observed. The above results provide a foundation for subsequent antioxidant research.

Intestinal barrier structure plays an important role in maintenance of normal physiological functions^29^. Tight junction (TJ) is an important connection mode between intestinal mucosal cells. TJ is a multifunctional complex composed of multiple proteins, mainly transmembrane proteins (Claudins, Occludin) and cytoplasmic proteins (ZO-1). When transmembrane proteins and cytoplasmic proteins are degenerated and damaged or synthesized insufficiently, TJ will be damaged and the intercellular permeability will be significantly increased, leading to the entry of macromolecules such as intestinal bacteria into the circulation (metabolic endotoxaemia)^30^. The results of this study found that H_2_O_2_ treatment of Caco-2 cells could cause a decrease expression of Claudin-1, Occludin and ZO-1, which was also reported by Somrudee et al.^31^. Compared with H_2_O_2_ stressed model, selenium-enriched C-1 could significantly increase expression of Claudin-1 and Occludin, not ZO-1. Somrudee et al. found that H_2_O_2_ can cause the breakdown of tight junctions, then increased expression of ZO-1 and Occludin^31^.

Safety for the intestinal cells, higher free radical scavenging ability, obvious anti-inflammatory activity, those characters are all pointed to the potential development of this bacteria. In order to investigate the mechanism of C-1 metabolizing Na_2_SeO_3_, transcriptome sequencing was used to analyze the functional classification and key pathways enriched by DEGs. ATP synthetase plays a central role in oxidative phosphorylation. Bacterial ATP synthetase is composed of hydrophobic domain F0 in the membrane and soluble hydrophilic head F1 outside the membrane. *atpA*, *atpD* and *atpG* genes encode α3, β3 and γ subunits of F1, respectively. Long et al. showed that under the stress of manganese, the overexpression of *atpA* gene could increase the number of bacteria logarithmically, and *atpD* gene was also positively regulated, participating in the stress response^32^. Our study showed that selenium-enriched C-1 was significantly enriched and down-regulated in the GO term associated with the protein complex during the vegetative stage, and *atpA*, *atpD*, *rplD*, *rplB*, *rplP*, *rplN*, and *rpsC* genes all showed down expression. Bacterial chemotaxis is the biased movement of bacteria toward a beneficial chemical gradient or away from a toxic chemical gradient by flagella. Quan et al. constructed *fliG* gene knockout mutant of *Campylobacter jejuni* NCTC11168, the chemotaxis and colonization ability of mutant were reduced, indicating that *fliG* gene was necessary in colonization chemotaxis for *C. jejuni*. FliM forms the C ring of flagellar matrix and participates in changing the direction of flagellar rotation^33^. The results of this study showed that in the sporulation stage, bacterial chemotaxis (bao02030) was down-regulated, and the GO term related to bacterial chemotaxis was significantly enriched and flagella-related genes (*flgG, fliM, fliL, fliJ*) were down-regulated in selenium-enriched C-1. It is hypothesized that *B. amyloliquefaciens* C-1 has reduced its motility, chemotaxis and colonization ability in a lower ATP consumption during the sporulation stage in response to selenium.

With the verified performance of SeNPs prepared by reduction of probiotic *B. amyloliquefaciens* C-1, the complex of *Bacillus* and SeNPs may be developed as postbiotics, which can be developed as food products with bacterial probiotics and postbiotics in great opportunity for research in food science, medicine, and nutrition, as well as in the food industry.

And there still exists limitations in this study, in the next step, it needs to carry out the safety evaluation at the animal model firstly, construct a successful and reliable oxidative stress animal model and verify the effectiveness of selenium-enriched C-1, which is of great significance for studying the mechanism. For the preparation of postbiotics, it is very important to process fermentation products and inactivate cells and retain effective active ingredients, which will increase our understanding of the health improvements of mineral-enriched postbiotics, including antioxidant functions, highlighting their perspective on microbial therapy to prevent and threaten gut-related diseases.

## Conclusions

*B. amyloliquefaciens* C-1, a patented strain, could convert Na_2_SeO_3_ into SeNPs by incubation with 100 μg/ml Na_2_SeO_3_ for 30 h fermentation at 30 °C with bio-conversion rate up to 55.51% per OD. Selenium-enriched C-1 had the high potentials to be developed as a postbiotics with good performances displayed in antioxidant, antibacterial, anti-inflammatory and protect the integrity of intestinal epithelium. During the process of SeNPs synthesis, the production capacity and metabolism of C-1 cells at vegetative stage slowed down, and motility decreased. At sporulation stage, a large amount of Na_2_SeO_3_ was transported inside of cell and the synthesized SeNPs accumulated intracellular. The metabolism of Na_2_SeO_3_ by C-1 can be used for the development of bio-SeNPs and postbiotics, and it may also be used as the bioremediation of heavy metal pollution in the environment.

## Materials and methods

### Bacterial strains and cell culture

*B. amyloliquefaciens* C-1 was a patented strain isolated from the ready-to-eat fruit samples at the Microbiology Lab of the Nutrition and Food Safety Engineering Research Center of Shaanxi province, Xi’an Jiaotong University, and stored in China Center for Type Culture Collection (CCTCC NO. M2012177). And the genome sequence of C-1 was submitted into NCBI with accession number of SRP127533.

C-1 is inoculated and cultured at LB + 1%Glucose (LB + G) medium and incubated aerobically at 30 °C. *Salmonella typhimurium* ATCC14028 used to stress the Caco-2 cells was stored at our lab, and cultured at LB medium at 37 °C.

Human-derived intestinal epithelial cells Caco-2 were used as an in vitro tested model for C-1 probiotics and cultured in RMPI 1640 medium (containing 20% fetal bovine serum, 1% antibiotics) in a 5% CO_2_ incubator at 37 °C. After the cells reached confluency, they were used in all downstream experimental analysis.

### The optical fermentation condition for reduction of selenite (SeO_3_^2−^)

To determine the optical selenite reduction and Selenium-enriched culture conditions for C-1, 1, 3, 5, 7, and 10% fresh overnight cultures of C-1 cells were sub-cultured into 250 mL Erlenmeyer flasks containing 100 ml LB + G medium with 0, 15, 30, 60, 100, and 150 μg/ml Na_2_SeO_3_, separately. The bacterial cultures were incubated at 30 °C on an orbital shaker at 200 rpm. Selenite in the medium and elemental Se in bacterial cells were determined through spectrophotometric methods as described^34^.

After confirmation of the optical subculture percentage and the supplied amount of Na_2_SeO_3_ in fermentation medium, the selenium-enriched growth of C-1 was monitored by measuring the optical density at 600 nm every 60 min at 30 °C for 22 h by a microplate reader (Tecan Infinite M200, Switzerland). Values obtained were expressed as rates of selenite reduction and elemental Se formation per OD_600_.

### Characterization of SeNPs produced

The OD_600_ of C-1 overnight culture was adjusted to 1.0, and a 10% subculture was transferred to a flask containing 100 ml of LB + G medium supplied with 100 μg/ml Na_2_SeO_3_, and incubated at 30 °C for 30 h. The cells were collected by centrifugation at 10,000 g for 15 min at 4 °C and washed 3 times with PBS. For morphological analysis, the collected C-1 cells were fixed with 2% glutaraldehyde and transferred to the Instrumental Analysis Center of Xi'an Jiaotong University for SEM (Hitachi SU3500, Japan), EDS (ThermoFisher ESCALAB Xi+ EDX, UK) and TEM (ThermoFisher Talos L120C, US) analysis. EDS combined with SEM can be used to analyze the types of elements in tested materials. For the detection of the elements contents within the selenium-enriched C-1 cells, cells were dried at 45 °C to a constant weight, and analyzed with XPS (Thermo Fisher ESCALAB Xi+ XPS, UK) in the Instrumental Analysis Center of Xi’an Jiaotong University.

### Free radical scavenging activity

To evaluate the ability of selenium-enriched *B. amyloliquefaciens* C-1 to resist oxidative stress, an in vitro scavenging free radical activity assay was conducted, which including superoxide anion(O_2_^−^), hydroxyl(OH^−^) and DPPH radicals by kits (catalog number as A052-1-1, A018-1-1, A153-1-1, Nanjing Jiancheng Institute of Bioengineering, Nanjing, China). Firstly, the bacterial suspension was centrifuged and resuspended using sterilized double-distilled water. The concentrations were adjusted to 2.5, 5, 10, 20, and 30 mg/ml. Then, the kit instructions were followed as manufacturer suggested. The suggested optical density measurement for detection of O_2_^−^, OH^−^ and DPPH radicals are 550, 550 and 517 nm, separately. The selenium-enriched C-1 and C-1 were the tested sample, as the ddH_2_O was set as blank control^35^.

The results were expressed as scavenging activity as follows.

$${\text{Scavenging}}\;{\text{activity}}\left( \% \right) = \left[ {\left( {{\text{A}}_{0} - {\text{A}}_{{1}} } \right)/{\text{A}}_{0} } \right] \times {1}00\%$$

A_0_: OD for the blank control, A_1_: OD for the tested sample.

### Anti-inflammation study using Caco-2 cells

#### Measurement of cell viability by MTT

Whole fermentation of C-1 and selenium-enriched C-1 cultures were diluted with RMPI 1640 medium to adjust bacterial cells concentrations to 8 × 10^6^, 1 × 10^6^, 8 × 10^5^, 1 × 10^5^, 8 × 10^4^, 1 × 10^4^, 8 × 10^3^ CFU/ml. Caco-2 cells (2.5 × 10^4^ cells/well) were seeded into 96-well plates overnight. Then cells were incubated with different concentration of C-1 and selenium-enriched C-1 for 20 h. After treatment, cells were washed twice with PBS and incubated with a 5 mg/ml MTT working solution for 4 h at 37 °C. Then, the supernatant was removed, and the culture was re-suspended in 150 μl of DMSO to dissolve the MTT formazan crystals, followed by mixing for 15 min. The OD_490_ was measured at a microplate reader (Tecan Infinite M200, Switzerland). The effect of C-1 and the selenium-enriched C-1 culture on Caco-2 cell viability was assessed as the percentage of viable cells in each treatment group relative to the untreated cells, and were arbitrarily assigned a viability of 100%.

$${\text{Cell}}\;{\text{viability}}\;{\text{rate}}\left( \% \right) = \left( {{\text{B}}_{{1}} /{\text{B}}_{{2}} } \right) \times {1}00$$

B_1_: OD_490_ for C-1 treatment or selenium-enriched C-1 treatment, B_2_: OD_490_ for negative control group.

#### Measurement of cell membrane integrity affected by* S. typhimurium*

Lactate dehydrogenase (LDH) cytotoxicity was assayed in Caco-2 cells culture medium to show the cell membrane integrity. Higher LDH activity means the damage of cell membrane and lower cell membrane integrity^36^. The method was used to measure cell viability and performed as described previously^26^. Caco-2 cells were seeded into 24-well plates, and cultivated to a single layer, then 4 treatment groups were set as C-1+S (2 h incubation with 1 × 10^7^ CFU/mL C-1 culture, then 1 × 10^7^ CFU/ml *S. typhimurium* ATCC14028 added and incubated for another 2 h); S+C-1 (2 h incubation with 1 × 10^7^ CFU/ml *S. typhimurium* ATCC14028 culture, then 1 × 10^7^ CFU/ml C-1 added and incubated for another 2 h); C-1-Se+S (2 h incubation with 1 × 10^7^ CFU/ml selenium-enriched C-1 culture, then 1 × 10^7^ CFU/ml *S. typhimurium* ATCC14028 added and incubated for another 2 h); S-C-1+Se (2 h incubation with 1 × 10^7^ CFU/ml *S. typhimurium* ATCC14028 culture, then 1 × 10^7^ CFU/ml selenium-enriched C-1 added and incubated for another 2 h). Caco-2 cells without any treatment was used as control. After incubation, the supernatant of cell culture was collected after centrifuging at 1500 rpm for 10 min at 4 °C. The released LDH into the culture supernatant through damaged membranes was measured spectrophotometrically using LDH Cytotoxicity Assay Kit (Nanjing Jiancheng Technology Co., Ltd., Nanjing, China), according to the manufacturer’s protocol.

#### Measurement of cell viability stressed by H_2_O_2_

Caco-2 cells (2.5 × 10^4^ cells per well) were seeded into 96-well plates overnight to a single layer. cells were pretreated with C-1 and selenium-enriched C-1 (1 × 10^5^, 8 × 10^4^, 1 × 10^4^, 8 × 10^3^ CFU/ml) for 20 h, then 200 μM H_2_O_2_ was added and incubated together for 4 h. Finally, the MTT assay and the survival rate were calculated according to method 5.5.1.

#### Measurement of cell cytokines and membrane permeability related protein genes by q-RT-PCR

To verify the protect effect of selenium-enriched C-1 on Caco-2 cells stressed byH_2_O_2_, the transcription of cell inflammatory cytokines (IL-8, IL-1β and TNF-ɑ), the membrane permeability protein genes (transmembrane protein gene Claudin-1, peripheral membrane protein gene ZO-1, Occludin) were detected q-RT-PCR^37^. Cell RNA extraction and relative mRNA expression of IL-8, IL-1β, TNF-α, ZO-1, clautin-1 and Occludin were determined according to the instructions of corresponding kits (Sigma-Aldrich). GAPDH was selected as the internal reference gene, and the relative mRNA expression levels were calculated according to 2^−ΔΔCT^. The primers used are provided in Table S1. The untreated Caco-2 cells was used as control, and all tests were performed in triplicate.

### Transcriptome analysis of the selenium-enriched metabolism of C-1

The transcriptome analysis of RNA-seq was performed as described previously^26^. Briefly, RNA was extracted separately from *B. amyloliquefaciens* C-1 grown in LB + G medium containing 100 μg/ml Na_2_SeO_3_ at the vegetative stage (16–18 h) and sporulation stage (36 h) using a bacteria RNA kit (Tiangen Biotech Co. Ltd, Beijing, China). C-1 grown in LB + G medium without Na_2_SeO_3_ was used as negative control, the control and treatment were all in triplicates. A total amount of 3 μg RNA per sample was used. Sequencing libraries were generated using NEBNext® UltraTM RNA Library Prep Kit for Illumina® (NEB, USA) following manufacturer’s recommendations and bar codes were added to attribute sequences of each sample. cDNA library was constructed and transcriptome sequencing was performed at Novogen Co. Ltd (Beijing, China). The raw data passed quality control and mapped to the reference genome of C-1 (SRP127533), differential expression analysis of vegetative stage (selenium-enriched C-1 vs. C-1), sporulation stage (selenium-enriched C-1 vs. C-1) was performed using the DESeq R package (v1.18.0). The resulted *P* values were adjusted using the Benjamini and Hochberg’s approach for controlling the false discovery rate (FDR). Genes with an adjusted *P* value < 0.05 found by DESeq were assigned as differentially expressed. FDR of 0.005 and log2(Fold change) of 1 were set as the threshold for significantly differential expression. For the differentially expressed genes, the GO (Gene Ontology) and KEGG (Kyoto Encyclopedia of Genes and Genomes) enrichment were analyzed^38^.

To validate the RNA-seq analysis data, q-RT-PCR was used to quantify gene expression levels of *B. amyloliquefaciens* C-1 cells with and without Se-enrichment under the same conditions used for RNA-seq analysis. Then reverse transcription and Quantitative reverse transcription polymerase chain reaction were performed according to the methods in 2.6.4. 16S rRNA was used as the internal reference. Three genes were randomly selected and the detail information of the primers for the selected genes were all listed in Table S1.

### Statistical analysis

All experimental data are shown as means ± SEM, and statistically analyzed by SPSS 22.0 (IBM Inc., IL, US). The statistical significance was calculated by one-way ANOVA and differences were significant at* P* < 0.05. And GraphPad Prism 7 was applied for graphical plotting and analysis.

### Supplementary Information

Supplementary Information.

## Data Availability

The data present in this study are available upon reasonable request from the corresponding author (hanbei@mail.xjtu.edu.cn).

## References

[1] Kieliszek M (2019). Selenium-fascinating microelement, properties and sources in food. Molecules.

[2] Gać P (2021). The importance of selenium and zinc deficiency in cardiovascular disorders. Environ. Toxicol. Pharmacol..

[3] Yang L (2022). Selenium concentration is associated with occurrence and diagnosis of three cardiovascular diseases: A systematic review and meta-analysis. J. Trace Elem. Med. Biol..

[4] Zhang L, Gao Y, Feng H, Zou N, Wang K, Sun D (2019). Effects of selenium deficiency and low protein intake on the apoptosis through a mitochondria-dependent pathway. J. Trace Elem. Med. Biol..

[5] Avery JC, Hoffmann PR (2018). Selenium, selenoproteins, and immunity. Nutrients.

[6] Nazıroğlu M, Öz A, Yıldızhan K (2020). Selenium and neurological diseases: Focus on peripheral pain and TRP channels. Curr. Neuropharmacol..

[7] Leiter O (2022). Selenium mediates exercise-induced adult neurogenesis and reverses learning deficits induced by hippocampal injury and aging. Cell Metab..

[8] Zhang S, Li B, Luo K (2022). Differences of selenium and other trace elements abundances between the Kaschin-Beck disease area and nearby non-Kaschin-Beck disease area, Shaanxi Province. China. Food Chem..

[9] Pieniz S, Andreazza R, Mann MB, Camargo F, Brandelli A (2017). Bioaccumulation and distribution of selenium in *Enterococcus durans*. J. Trace Elem. Med. Biol..

[10] Ali HFH, El-Sayed NM, Khodeer DM, Ahmed AAM, Hanna PA, Moustafa YMA (2020). Nano selenium ameliorates oxidative stress and inflammatory response associated with cypermethrin-induced neurotoxicity in rats. Ecotoxicol. Environ. Saf..

[11] Abedi S, Iranbakhsh A, Oraghi AZ, Ebadi M (2021). Nitric oxide and selenium nanoparticles confer changes in growth, metabolism, antioxidant machinery, gene expression, and flowering in chicory (*Cichorium intybus* L.): Potential benefits and risk assessment. Environ. Sci. Pollut. Res. Int..

[12] Geoffrion LD (2020). Naked selenium nanoparticles for antibacterial and anticancer treatments. ACS Omega.

[13] Shi XD, Tian YQ, Wu JL, Wang SY (2021). Synthesis, characterization, and biological activity of selenium nanoparticles conjugated with polysaccharides. Crit. Rev. Food Sci. Nutr..

[14] Srivastava N, Mukhopadhyay M (2015). Green synthesis and structural characterization of selenium nanoparticles and assessment of their antimicrobial property. Bioprocess Biosyst. Eng..

[15] Kim SK (2019). Role of probiotics in human gut microbiome-associated diseases. J. Microbiol. Biotechnol..

[16] Borchers AT, Selmi C, Meyers FJ, Keen CL, Gershwin ME (2009). Probiotics and immunity. J. Gastroenterol..

[17] Vera-Santander VE, Hernández-Figueroa RH, Jiménez-Munguía MT, Mani-López E, López-Malo A (2023). Health benefits of consuming foods with bacterial probiotics, postbiotics, and their metabolites: A review. Molecules.

[18] Shoeibi S, Mashreghi M (2017). Biosynthesis of selenium nanoparticles using *Enterococcus faecalis* and evaluation of their antibacterial activities. J. Trace Elem. Med. Biol..

[19] Beleneva IA (2022). Biogenic synthesis of selenium and tellurium nanoparticles by marine bacteria and their biological activity. World J. Microbiol. Biotechnol..

[20] Hashem AH (2021). *Bacillus megaterium*-mediated synthesis of selenium nanoparticles and their antifungal activity against *Rhizoctonia solani* in faba bean plants. J. Fungi.

[21] Akçay FA, Avcı A (2020). Effects of process conditions and yeast extract on the synthesis of selenium nanoparticles by a novel indigenous isolate *Bacillus sp.* EKT1 and characterization of nanoparticles. Arch. Microbiol..

[22] Dhanjal S, Cameotra SS (2010). Aerobic biogenesis of selenium nanospheres by *Bacillus cereus* isolated from coalmine soil. Microb. Cell Fact..

[23] Ashengroph M, Hosseini SR (2021). A newly isolated *Bacillus amyloliquefaciens* SRB04 for the synthesis of selenium nanoparticles with potential antibacterial properties. Int. Microbiol..

[24] Fischer S (2020). *Bacillus safensis* JG-B5T affects the fate of selenium by extracellular production of colloidally less stable selenium nanoparticles. J. Hazard Mater..

[25] Cheng Y (2017). Characterization, antioxidant property and cytoprotection of exopolysaccharide-capped elemental selenium particles synthesized by *Bacillus paralicheniformis* SR14. Carbohydr. Polym..

[26] Liu J (2022). Preparation, characteristic and anti-inflammatory effect of selenium nanoparticle-enriched probiotic strain *Enterococcus durans* A8–1. J. Trace Elem. Med. Biol..

[27] Shakibaie M (2017). Probiotic and antioxidant properties of selenium-enriched *Lactobacillus brevis* LSe isolated from an Iranian traditional dairy product. J. Trace Elem. Med. Biol..

[28] Xu C, Guo Y, Qiao L, Ma L, Cheng Y, Roman A (2018). Biogenic synthesis of novel functionalized selenium nanoparticles by *Lactobacillus casei* ATCC 393 and its protective effects on intestinal barrier dysfunction caused by enterotoxigenic *Escherichia coli* K88. Front. Microbiol..

[29] Hossain Z, Hirata T (2008). Molecular mechanism of intestinal permeability: Interaction at tight junctions. Mol. Biosyst..

[30] Zhao X (2021). Tight junctions and their regulation by non-coding RNAs. Int. J. Biol. Sci..

[31] Chuenkitiyanon S, Pengsuparp T, Jianmongkol S (2010). Protective effect of quercetin on hydrogen peroxide-induced tight junction disruption. Int. J. Toxicol..

[32] Long H, Niu X, Huang S, Ran X, Wang J (2020). Response of genes related to oxidative phosphorylation pathway of *Bacillus safensis* under manganese stress. J. Ind. Microbiol..

[33] Quan S, Xia X, Zhao X, Luo Y, Yan J (2009). Alterations of motility and chemotaxis in vitro and colonization in mice of *fliG* gene knockout mutant of *Campylobacter jejuni*. J. Microbes Infect..

[34] Biswas KC, Barton LL, Tsui WL, Shuman K, Gillespie J, Eze CS (2011). A novel method for the measurement of elemental selenium produced by bacterial reduction of selenite. J. Microbiol. Methods.

[35] Wang F (2022). Exopolymer-functionalized nanoselenium from *Bacillus subtilis* SR41: Characterization, monosaccharide analysis and free radical scavenging ability. Polymers.

[36] Cummings BS, Schnellmann RG (2021). Measurement of cell death in mammalian cells. Curr. Protoc..

[37] Zhou Y (2021). Probiotic potential analysis and safety evaluation of *Enterococcus durans* A8-1 isolated from a healthy Chinese infant. Front. Microbiol..

[38] Kanehisa M, Goto S (2000). KEGG: Kyoto encyclopedia of genes and genomes. Nucleic Acids Res..

