# Supplementary material for: Characterization and anti-inflammatory effect of selenium-enriched probiotic Bacillus amyloliquefaciens C-1, a potential postbiotics
Source: Sci Rep. 2023 Aug 31;13:14302. doi: 10.1038/s41598-023-40988-8 (PMC10471622; doi:10.1038/s41598-023-40988-8)
Supplement: Supplementary file 1 — Supplementary Information. [file 41598_2023_40988_MOESM1_ESM.docx]

**Supplementary information**

**FigureS1** Correlation of fold change analyzed by data obtained using RNA-Seq platform with q-RT-PCR.

**TableS1** Primers list of q-RT-PCR.

**TableS2** Differentially expressed genes in Se-enriched *B. amyloliquefaciens* C-1 vs *B. amyloliquefaciens* C-1(|log2FoldChange|＞2, *P_adj_*＜0.05).

**TableS3** The Gene Ontology (GO) functional annotation of differentially expressed genes Se-enriched *B. amyloliquefaciens* C-1 (BP, Biological Process; CC, cellular Component; MF, Molecular Function).

**TableS4** Differentially expressed genes by KEGG pathway categories.

**TableS5** Differential genes associated with ABC transporters.

**FigureS1** Correlation of fold change analyzed by data obtained using RNA-Seq with q-RT-PCR

**
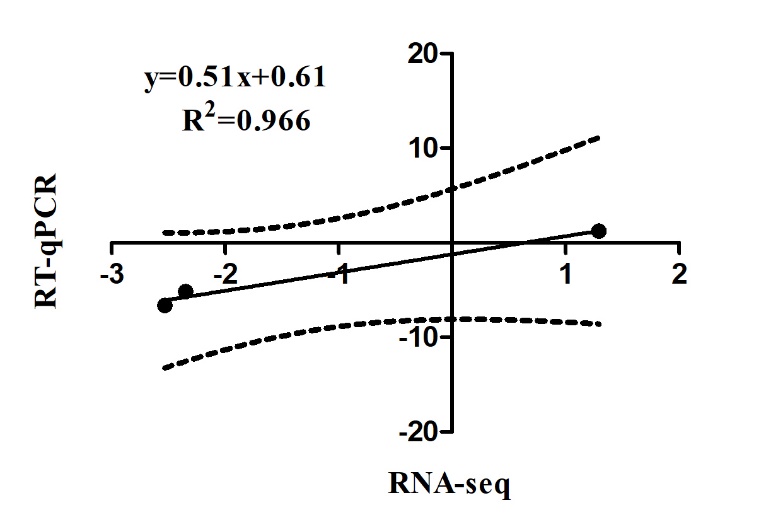
**

**Table S1** Primers list of Q-RT-PCR

| Genes | Genes name | Primer sequence（5'→3'） | Amplified fragments （bp） | Annealing temperature (℃) |
| --- | --- | --- | --- | --- |
| IL-1β | IL-1β-F | GAATGACGCCCTCAATCAAAGT | 186 | 55 |
|  | IL-1β-R | TCATCTTGGGCAGTCACATACA |  |  |
| IL-8 | IL-8-F | CCTGAACCTTCCAAAGATGGC | 75 | 55 |
|  | IL-8-R | TTCACCAGGCAAGTCTCCTCA |  |  |
| TNF-α | TNF-α-F | GAGGCCAAGCCCTGGTATG | 91 | 56 |
|  | TNF-α-R | CGGGCCGATTGATCTCAGC |  |  |
| Occludin | Occludin-F | CTTCCAATGGCAAAGTGAATGAATGA | 67 | 57 |
|  | Occludin-R | TACCACCGCTGCTGTAACGAG |  |  |
| claudin | Claudin-F | CCAGGTACGAATTTGGTCAGG | 119 | 54 |
|  | Claudin-R | TGGTGTTGGGTAAGAGGTTGT |  |  |
| ZO-1 | ZO-1-F | GAGCCTAATCTGACCTATGAACC | 93 | 54 |
|  | ZO-1-R | TGAGGACTCGTATCTGTATGTGG |  |  |
| GAPDH | GAPDH-F | CCATTTGATGTTAGCGGGATCTC | 134 | 54 |
|  | GAPDH-R | TGGTCTACATGTTCCAGTATGACT |  |  |
| RS17905  (*atpD*) | RS17905-F | CATCACCGACAGGAACGGAA | 168 | 55 |
|  | RS17905-R | AGCCAGCTACAGGCGAAAAT |  |  |
| RS07005  (*pfkA*) | RS07005-F | CCCTCATTACGGACGACCTG | 80 | 54 |
|  | RS07005-R | CTCGCTGATTTCAGGCTTGC |  |  |
| RS13550  (putative gene) | RS13550-F | ACACCTACACACGGAAACCC | 82 | 55 |
|  | RS13550-R | AAGGTCTTGTCGTCATCGGC |  |  |
| 16sRNA | 16sRNA-F | CCTACGGNGGCWGCAG | 464 | 52 |
|  | 16sRNA-R | GACTACHVGGGTATCTAATCC |  |  |

**Table S2 Sample sequencing data quality summary.**

| Sample name | Raw reads | Clean reads | Clean bases | Error rate(%） | Q20  （%） | Q30  （%） | GC content（%） |
| --- | --- | --- | --- | --- | --- | --- | --- |
| C1_A_1 | 7435774 | 7406752 | 1.12G | 0.03 | 97.94 | 94.09 | 48.42 |
| C1_A_2 | 7898918 | 7866628 | 1.18G | 0.03 | 97.9 | 94 | 48 |
| C1_A_3 | 8094648 | 8062636 | 1.21G | 0.03 | 97.77 | 93.7 | 47.49 |
| C1_A_Se_1 | 7807578 | 7760488 | 1.17G | 0.03 | 97.9 | 94.01 | 47.35 |
| C1_A_Se_2 | 7720310 | 7679390 | 1.16G | 0.03 | 97.77 | 93.72 | 47.91 |
| C1_A_Se_3 | 7657214 | 7627852 | 1.15G | 0.03 | 97.94 | 94.08 | 47.01 |
| C1_B_1 | 7553326 | 7477504 | 1.13G | 0.02 | 98.06 | 94.33 | 47.94 |
| C1_B_2 | 7316888 | 7258202 | 1.09G | 0.03 | 97.88 | 93.96 | 47.25 |
| C1_B_3 | 7837936 | 7775142 | 1.17G | 0.02 | 98.09 | 94.36 | 47.62 |
| C1_B_Se_1 | 7765610 | 7726586 | 1.16G | 0.02 | 98.7 | 95.84 | 47.01 |
| C1_B_Se_2 | 7622402 | 7555624 | 1.14G | 0.03 | 97.97 | 94.13 | 47.13 |
| C1_B_Se_3 | 7109798 | 7025982 | 1.06G | 0.02 | 98.38 | 95.21 | 47.1 |

Note:

C1_A_1, C1_A_2, C1_A_3 represent the results of three repeated experiments of C-1 at vegetative stage; C1_A_Se_1, C1_A_Se_2, C1_A_Se_3 represent the results of three repeated experiments of selenium-enriched C-1 at vegetative stage.

C1_B_1, C1_B_2, C1_B_3 represent the results of three repeated experiments of C-1 at spores stage; C1_B_Se_1, C1_B_Se_2, C1_B_Se_3 represent the results of three repeated experiments of selenium-enriched C-1 at spores stage.

**TableS3** Differentially expressed genes in Se-enriched *B. amyloliquefaciens* C-1 vs *B. amyloliquefaciens* C-1 (log2FoldChange>2, *P_adj_*<0.05).

| Stage | Gene ID | Description | log2FC | P_adj_ |
| --- | --- | --- | --- | --- |
| Vegetative stage | RS04830 | alpha/beta-type small acid-soluble spore protein | +5.7591 | <0.001 |
|  | RS16610 | GntP family permease | +3.6857 | <0.001 |
|  | RS18805 | ROK family protein | +3.5357 | <0.001 |
|  | RS03820 | Maltogenic Amylase, C-terminal domain | +3.5211 | <0.001 |
|  | RS18810 | Phosphomannose isomerase type I | +3.4591 | <0.001 |
|  | RS06320 | Phage terminase small subunit | +3.4102 | <0.001 |
|  | RS16605 | gluconokinase | +3.3415 | <0.001 |
|  | RS18795 | PTS cellobiose transporter subunit IIC | +3.1614 | <0.001 |
|  | RS01170 | LrgB family protein | +3.1379 | 0.0020 |
|  | RS18815 | Glycosyl hydrolase family 26 | +3.0969 | <0.001 |
|  | RS06360 | hypothetical protein | +2.9584 | 0.0008 |
|  | RS18800 | Glycosyl hydrolase family 1 | +2.9106 | <0.001 |
|  | RS06325 | PBSX family phage terminase large subunit | +2.9017 | 0.0004 |
|  | RS18120 | Nitrate reductase gamma subunit | +2.8190 | 0.0010 |
|  | RS15025 | Na+ dependent nucleoside transporter N-terminus | +2.8030 | 0.0004 |
|  | RS06860 | ATP-dependent Clp protease ATP-binding subunit | +2.7965 | 0.0028 |
|  | RS06335 | hypothetical protein | +2.6997 | <0.001 |
|  | RS04530 | cold-shock protein | +2.6988 | 0.0052 |
|  | RS07345 | hypothetical protein | +2.6536 | 0.0015 |
|  | RS18820 | catalase | +2.6367 | 0.0010 |
|  | RS02175 | D-lyxose isomerase | +2.6081 | 0.0023 |
|  | RS06380 | phage portal protein | +2.6069 | 0.0004 |
|  | RS04670 | mechanosensitive ion channel | +2.5783 | 0.0010 |
|  | RS13680 | alpha/beta-type small acid-soluble spore protein | +2.5366 | 0.0088 |
|  | RS18790 | PTS lactose/cellobiose transporter subunit IIA | +2.4640 | 0.0001 |
|  | RS11465 | alpha/beta fold hydrolase | +2.4375 | <0.001 |
|  | RS03815 | PTS system trehalose-specific EIIBC component | +2.4329 | 0.0001 |
|  | RS06445 | hypothetical protein | +2.4316 | 0.0049 |
|  | RS03825 | trehalose operon repressor | +2.3839 | 0.0002 |
|  | RS02300 | general stress protein | +2.3745 | 0.0004 |
|  | RS03260 | hypothetical protein | +2.3719 | <0.001 |
|  | RS06330 | phage portal protein | +2.3539 | 0.0002 |
|  | RS17750 | ammonium transporter | +2.3500 | 0.0017 |
|  | RS06400 | hypothetical protein | +2.3073 | 0.0012 |
|  | RS02550 | BCCT family transporter | +2.2852 | 0.0004 |
|  | RS03185 | sigma-70 family RNA polymerase sigma factor | +2.2336 | 0.0059 |
|  | RS18785 | PTS sugar transporter subunit IIB | +2.2160 | <0.001 |
|  | RS02990 | TetR family transcriptional regulator | +2.2152 | 0.0026 |
|  | RS02170 | SDR family oxidoreductase | +2.1883 | 0.0015 |
|  | RS19715 | 3-hydroxybutyrate dehydrogenase | +2.1822 | 0.0368 |
|  | RS18130 | nitrate reductase subunit beta | +2.1769 | 0.0119 |
|  | RS04815 | ABC transporter | +2.1712 | 0.0040 |
|  | RS06450 | hemolysin XhlA family protein | +2.1533 | 0.0134 |
|  | RS11215 | transcriptional repressor | +2.1207 | 0.0088 |
|  | RS15800 | YvrJ family protein | +2.1197 | 0.0014 |
|  | RS16560 | gapA transcriptional regulator CggR | +2.0988 | 0.0066 |
|  | RS15950 | cadmium-translocating P-type ATPase | +2.0951 | 0.0015 |
|  | RS02600 | metal-sensitive transcriptional regulator | +2.0810 | 0.0269 |
|  | RS06355 | HK97 gp10 family phage protein | +2.0477 | 0.0087 |
|  | RS13145 | ribonuclease PH | +2.0264 | 0.0314 |
|  | RS05650 | thiol management oxidoreductase | +2.0223 | 0.0368 |
|  | RS07050 | DUF1797 family protein | +2.0137 | 0.0237 |
|  | RS15570 | acyl-CoA dehydrogenase family protein | -7.9031 | <0.001 |
|  | RS07680 | S8 family serine peptidase | -7.4537 | <0.001 |
|  | RS03985 | dihydrolipoyl dehydrogenase | -7.2566 | <0.001 |
|  | RS03980 | e3 binding domain | -7.0734 | <0.001 |
|  | RS15575 | acetyl-CoA C-acetyltransferase | -6.8428 | <0.001 |
|  | RS03975 | Transketolase, C-terminal domain | -6.6687 | <0.001 |
|  | RS13215 | Electron transfer flavoprotein domain | -5.8396 | <0.001 |
|  | RS15580 | Enoyl-CoA hydratase/isomerase | -5.7843 | <0.001 |
|  | RS03970 | Dehydrogenase E1 component | -5.5615 | <0.001 |
|  | RS09735 | glucosylceramidase | -5.4867 | <0.001 |
|  | RS01920 | amino acid ABC transporter substrate-binding protein | -5.3382 | <0.001 |
|  | RS01930 | ABC transporter | -5.1348 | <0.001 |
|  | RS09175 | 3-hydroxybutyrate dehydrogenase | -5.1124 | <0.001 |
|  | RS01925 | amino acid ABC transporter permease | -5.0475 | <0.001 |
|  | RS04000 | 6-phospho-alpha-glucosidase | -4.9310 | <0.001 |
|  | RS18085 | (Fe-S)-binding protein | -4.8950 | <0.001 |
|  | RS08890 | peptidase G2 | -4.8933 | <0.001 |
|  | RS01915 | GNAT family N-acetyltransferase | -4.8706 | <0.001 |
|  | RS01935 | M20 peptidase aminoacylase family protein | -4.7370 | <0.001 |
|  | RS13220 | Electron transfer flavoprotein domain | -4.7100 | <0.001 |
|  | RS09180 | CoA transferase subunit B | -4.7021 | <0.001 |
|  | RS09185 | CoA transferase subunit A | -4.5230 | <0.001 |
|  | RS13225 | enoyl-CoA hydratase | -4.5198 | <0.001 |
|  | RS08870 | lytic polysaccharide monooxygenase | -4.3870 | <0.001 |
|  | RS18730 | 6-phospho-beta-glucosidase | -4.2686 | <0.001 |
|  | RS01675 | hypothetical protein | -4.2098 | 0.0001 |
|  | RS05095 | AMP-binding enzyme C-terminal domain | -4.1465 | <0.001 |
|  | RS06010 | Glycosyl hydrolase family 53 | -4.1356 | <0.001 |
|  | RS12585 | Cys/Met metabolism PLP-dependent enzyme | -4.0907 | <0.001 |
|  | RS13455 | glyceraldehyde-3-phosphate dehydrogenase | -4.0249 | <0.001 |
|  | RS05110 | S8 family serine peptidase | -3.9980 | <0.001 |
|  | RS01910 | LLM class flavin-dependent oxidoreductase | -3.8046 | <0.001 |
|  | RS13230 | TetR/AcrR family transcriptional regulator | -3.7831 | <0.001 |
|  | RS09165 | non-ribosomal peptide synthetase | -3.7527 | <0.001 |
|  | RS09345 | gamma-glutamyltransferase | -3.7523 | <0.001 |
|  | RS07505 | cytochrome c oxidase assembly factor CtaG | -3.6966 | <0.001 |
|  | RS15915 | assimilatory sulfite reductase (NADPH) hemoprotein subunit | -3.6899 | <0.001 |
|  | RS07500 | cytochrome c oxidase subunit IVB | -3.6392 | <0.001 |
|  | RS04545 | L-cystine transporter | -3.5617 | <0.001 |
|  | RS08535 | glycine C-acetyltransferase | -3.5509 | <0.001 |
|  | RS07830 | adenylyl-sulfate kinase | -3.4127 | <0.001 |
|  | RS01055 | DUF1343 domain-containing protein | -3.3739 | <0.001 |
|  | RS04010 | PTS transporter subunit EIIC | -3.3678 | <0.001 |
|  | RS09160 | non-ribosomal peptide synthetase | -3.3579 | <0.001 |
|  | RS12500 | Phosphomannose isomerase type I | -3.3223 | <0.001 |
|  | RS18735 | PTS lactose/cellobiose transporter subunit IIA | -3.2639 | <0.001 |
|  | RS02275 | hypothetical protein | -3.2432 | <0.001 |
|  | RS15935 | zinc-dependent metalloprotease | -3.1926 | 0.0005 |
|  | RS15920 | assimilatory sulfite reductase (NADPH) flavoprotein subunit | -3.1831 | <0.001 |
|  | RS15830 | Periplasmic binding protein | -3.1794 | <0.001 |
|  | RS07495 | cytochrome (ubi)quinol oxidase subunit III | -3.1385 | <0.001 |
|  | RS01630 | starch-binding protein | -3.1324 | <0.001 |
|  | RS09145 | glucuronoxylanase xynC | -3.1220 | <0.001 |
|  | RS15815 | ABC transporter | -3.1220 | 0.0002 |
|  | RS12745 | 6S RNA | -3.1187 | 0.0028 |
|  | RS14160 | phosphoenolpyruvate carboxykinase (ATP) | -3.1002 | <0.001 |
|  | RS06245 | S9 family peptidase | -3.0888 | 0.0019 |
|  | RS12505 | PTS sugar transporter subunit IIA | -3.0793 | <0.001 |
|  | RS02000 | siderophore ABC transporter substrate-binding protein | -3.0603 | 0.0001 |
|  | RS02055 | DUF1775 domain-containing protein | -3.0258 | <0.001 |
|  | RS06025 | galactokinase | -2.9864 | <0.001 |
|  | RS01545 | zinc ABC transporter substrate-binding protein | -2.9840 | <0.001 |
|  | RS03315 | Phosphoribosyl transferase domain | -2.9839 | 0.0001 |
|  | RS16345 | transfer-messenger RNA | -2.9754 | 0.0015 |
|  | RS01140 | Glutamine amidotransferase domain | -2.9698 | <0.001 |
|  | RS01060 | Glycosyl hydrolase family 3 N terminal domain | -2.9282 | <0.001 |
|  | RS06655 | S8 family peptidase | -2.9228 | 0.0018 |
|  | RS06255 | N-acetylmuramoyl-L-alanine amidase | -2.8935 | 0.0003 |
|  | RS07390 | peptidase M4 family protein | -2.8773 | <0.001 |
|  | RS05055 | ketoacyl-ACP synthase III | -2.8537 | <0.001 |
|  | RS12580 | YrhC family protein | -2.8404 | <0.001 |
|  | RS06015 | :Galactose-1-phosphate uridyl transferase, C-terminal domain | -2.8121 | <0.001 |
|  | RS15640 | ABC transporter | -2.8024 | 0.0003 |
|  | RS01940 | MmgE/PrpD family | -2.7995 | 0.0004 |
|  | RS09155 | non-ribosomal peptide synthetase | -2.7977 | <0.001 |
|  | RS04015 | ABC transporter | -2.7603 | 0.0021 |
|  | RS19440 | Arginase family | -2.7406 | 0.0001 |
|  | RS07235 | gamma-glutamylcyclotransferase | -2.7177 | 0.0002 |
|  | RS07490 | cytochrome c oxidase subunit I | -2.7143 | 0.0001 |
|  | RS07630 | stage V sporulation protein D | -2.7061 | 0.0066 |
|  | RS01115 | RNA polymerase sigma factor SigW | -2.7028 | 0.0001 |
|  | RS03680 | pectate lyase | -2.6986 | <0.001 |
|  | RS09150 | carbohydrate-binding protein | -2.6958 | <0.001 |
|  | RS06020 | UDP-glucose 4-epimerase GalE | -2.6866 | <0.001 |
|  | RS01120 | anti-sigma-W factor RsiW | -2.6797 | 0.0001 |
|  | RS00130 | signal recognition particle sRNA large type | -2.6637 | 0.0034 |
|  | RS01050 | AraC family transcriptional regulator | -2.6574 | 0.0003 |
|  | RS17675 | single-stranded DNA-binding protein | -2.6549 | <0.001 |
|  | RS12240 | hypothetical protein | -2.6508 | 0.0003 |
|  | RS19120 | Arginase family | -2.6413 | 0.0003 |
|  | RS09190 | GntP family permease | -2.6387 | <0.001 |
|  | RS19445 | amino acid permease | -2.6381 | 0.0003 |
|  | RS03335 | phosphoribosylamine--glycine ligase | -2.6151 | 0.0002 |
|  | RS03845 | 5'-nucleotidase, C-terminal domain | -2.5622 | 0.0001 |
|  | RS16440 | Binding-protein-dependent transport system inner membrane component | -2.5484 | 0.0003 |
|  | RS18380 | Glu/Leu/Phe/Val dehydrogenase, dimerisation domain | -2.5480 | 0.0004 |
|  | RS08740 | Ribonucleotide reductase, small chain | -2.5416 | <0.001 |
|  | RS13450 | S-adenosylmethionine decarboxylase | -2.5305 | 0.0005 |
|  | RS18740 | PTS cellobiose transporter subunit IIC | -2.5093 | <0.001 |
|  | RS15165 | ribonuclease | -2.5056 | 0.0002 |
|  | RS07330 | Transketolase, pyrimidine binding domain | -2.4711 | 0.0008 |
|  | RS06650 | Cobalamin-independent synthase, Catalytic domain | -2.4655 | <0.001 |
|  | RS07825 | ATP-sulfurylase | -2.4467 | 0.0002 |
|  | RS18285 | DUF1700 domain-containing protein | -2.4393 | 0.0024 |
|  | RS10545 | RNase P RNA component class B | -2.4364 | 0.0043 |
|  | RS14415 | flotillin family protein | -2.4285 | 0.0002 |
|  | RS05600 | oligopeptide ABC transporter ATP-binding protein OppF | -2.4244 | 0.0002 |
|  | RS03325 | Formyl transferase | -2.3839 | 0.0099 |
|  | RS14435 | choline dehydrogenase | -2.3807 | <0.001 |
|  | RS03330 | AICARFT/IMPCHase bienzyme\|PF02142:MGS-like domain | -2.3758 | 0.0090 |
|  | RS12245 | SigmaW regulon antibacterial | -2.3633 | 0.0004 |
|  | RS11100 | riboflavin synthase | -2.3508 | <0.001 |
|  | RS19210 | Fructose-bisphosphate aldolase class-II | -2.3490 | 0.0003 |
|  | RS17905 | F0F1 ATP synthase subunit beta | -2.3486 | 0.0008 |
|  | RS19450 | Aminotransferase class-III | -2.3357 | 0.0016 |
|  | RS04145 | Bacterial SH3 domain | -2.3207 | 0.0002 |
|  | RS03310 | AIR synthase related protein, C-terminal domain | -2.3206 | 0.0035 |
|  | RS02945 | serine hydrolase | -2.3164 | 0.0005 |
|  | RS03420 | efflux RND transporter permease subunit | -2.3130 | 0.0001 |
|  | RS07985 | 3-oxoacyl-[acyl-carrier-protein] reductase | -2.3107 | 0.0002 |
|  | RS06535 | peptide ABC transporter substrate-binding protein | -2.3003 | 0.0009 |
|  | RS19115 | Amidohydrolase family | -2.2574 | 0.0005 |
|  | RS08880 | hypothetical protein | -2.2391 | 0.0010 |
|  | RS17030 | phage holin family protein | -2.2290 | 0.0001 |
|  | RS14205 | YtzI protein | -2.2231 | 0.0008 |
|  | RS09085 | mannonate dehydratase | -2.2043 | 0.0016 |
|  | RS06030 | PTS lactose transporter subunit IIBC | -2.2003 | 0.0009 |
|  | RS11090 | 6,7-dimethyl-8-ribityllumazine synthase | -2.1953 | <0.001 |
|  | RS18375 | Aldehyde dehydrogenase family | -2.1924 | 0.0003 |
|  | RS01065 | penicillin binding protein PBP4B | -2.1804 | 0.0004 |
|  | RS15820 | iron ABC transporter permease | -2.1802 | 0.0404 |
|  | RS17075 | TetR/AcrR family transcriptional regulator | -2.1735 | 0.0001 |
|  | RS18520 | S8 family serine peptidase | -2.1731 | 0.0095 |
|  | RS12980 | SPOR domain-containing protein | -2.1616 | 0.0025 |
|  | RS17070 | phosphotransferase | -2.1499 | <0.001 |
|  | RS01550 | ABC transporter | -2.1141 | 0.0006 |
|  | RS05175 | hypothetical protein | -2.1040 | 0.0016 |
|  | RS17865 | VWA domain-containing protein | -2.0916 | 0.0201 |
|  | RS02060 | copper resistance protein CopC | -2.0859 | 0.0029 |
|  | RS18945 | Glycosyl hydrolases family 16 | -2.0847 | 0.0017 |
|  | RS05580 | peptide ABC transporter substrate-binding protein | -2.0772 | 0.0008 |
|  | RS11035 | AhpC/TSA family | -2.0738 | 0.0098 |
|  | RS19295 | fatty acid desaturase | -2.0708 | 0.0087 |
|  | RS12250 | nodulation protein NfeD | -2.0647 | 0.0025 |
|  | RS19110 | urocanate hydratase | -2.0602 | 0.0010 |
|  | RS17900 | F0F1 ATP synthase subunit epsilon | -2.0577 | 0.0008 |
|  | RS04810 | universal stress protein | -2.0524 | 0.0017 |
|  | RS07485 | cytochrome c oxidase subunit II | -2.0505 | 0.0035 |
|  | RS08735 | class 1b ribonucleoside-diphosphate reductase subunit alpha | -2.0462 | 0.0008 |
|  | RS14595 | hydrolase | -2.0280 | 0.0004 |
|  | RS11095 | GTP cyclohydrolase II | -2.0229 | <0.001 |
|  | RS19125 | amino acid permease | -2.0228 | 0.0064 |
|  | RS10740 | menaquinol-cytochrome c reductase cytochrome b/c subunit | -2.0213 | 0.0004 |
|  | RS09585 | Aldehyde dehydrogenase family | -2.0207 | 0.0022 |
|  | RS01045 | ABC transporter substrate-binding protein | -2.0159 | 0.0192 |
|  | RS15155 | Aminotransferase class-V | -2.0158 | 0.0297 |
| Spores stage | RS03050 | tRNA-Asp | +5.9988 | <0.001 |
|  | RS13765 | cation transporter | +5.4799 | <0.001 |
|  | RS04005 | MurR/RpiR family transcriptional regulator | +5.4426 | <0.001 |
|  | RS16610 | GntP family permease | +4.7708 | <0.001 |
|  | RS16605 | gluconokinase | +4.5163 | <0.001 |
|  | RS17215 | competence protein ComFB | +4.4518 | <0.001 |
|  | RS14125 | glycerophosphoryl diester phosphodiesterase | +4.3063 | <0.001 |
|  | RS04000 | 6-phospho-alpha-glucosidase | +4.2991 | <0.001 |
|  | RS03185 | sigma-70 family RNA polymerase sigma factor | +4.2620 | <0.001 |
|  | RS08120 | flagellar basal body rod protein FlgB | +4.1986 | <0.001 |
|  | RS10620 | Holliday junction resolvase RecU | +4.1231 | <0.001 |
|  | RS17490 | acetolactate decarboxylase | +4.0222 | <0.001 |
|  | RS17750 | ammonium transporter | +3.9678 | <0.001 |
|  | RS18350 | bacilysin biosynthesis protein BacA | +3.9601 | <0.001 |
|  | RS06480 | DUF47 domain-containing protein | +3.9413 | <0.001 |
|  | RS04010 | PTS transporter subunit EIIC | +3.8876 | <0.001 |
|  | RS03260 | hypothetical protein | +3.8759 | <0.001 |
|  | RS02935 | hypothetical protein | +3.8275 | <0.001 |
|  | RS13370 | hypothetical protein | +3.7848 | <0.001 |
|  | RS14615 | general stress protein 13 | +3.7616 | <0.001 |
|  | RS18785 | PTS sugar transporter subunit IIB | +3.7208 | <0.001 |
|  | RS14625 | AsnC family | +3.6940 | <0.001 |
|  | RS01420 | cation transporter | +3.6720 | <0.001 |
|  | RS11465 | alpha/beta fold hydrolase | +3.6368 | <0.001 |
|  | RS18690 | GTP pyrophosphokinase family protein | +3.6359 | <0.001 |
|  | RS06695 | MerR HTH family regulatory protein | +3.6268 | <0.001 |
|  | RS08125 | flagellar basal body rod protein FlgC | +3.5844 | <0.001 |
|  | RS09605 | Sodium Bile acid symporter family | +3.5686 | <0.001 |
|  | RS04765 | CrcB family protein | +3.5522 | 0.0012 |
|  | RS01845 | amino acid ABC transporter permease | +3.5364 | <0.001 |
|  | RS12105 | transcriptional repressor | +3.5362 | <0.001 |
|  | RS00015 | ribosome maturation protein RlbA | +3.4795 | <0.001 |
|  | RS18800 | Glycosyl hydrolase family 1 | +3.4715 | <0.001 |
|  | RS05515 | Basic membrane protein | +3.4593 | <0.001 |
|  | RS12405 | HAD-hyrolase-like | +3.4464 | <0.001 |
|  | RS18795 | Phosphotransferase system, EIIC | +3.4291 | <0.001 |
|  | RS05970 | hypothetical protein | +3.4262 | 0.0001 |
|  | RS00250 | transition state genes transcriptional regulator AbrB | +3.4002 | <0.001 |
|  | RS06860 | ATP-dependent Clp protease ATP-binding subunit | +3.3845 | <0.001 |
|  | RS02990 | TetR family transcriptional regulator | +3.3596 | <0.001 |
|  | RS18790 | PTS lactose/cellobiose transporter subunit IIA | +3.3145 | <0.001 |
|  | RS18805 | ROK family protein | +3.2972 | <0.001 |
|  | RS02105 | LamB/YcsF family protein | +3.2655 | <0.001 |
|  | RS15950 | cadmium-translocating P-type ATPase | +3.2297 | <0.001 |
|  | RS07050 | DUF1797 family protein | +3.2155 | <0.001 |
|  | RS13780 | AzlD domain-containing protein | +3.2093 | <0.001 |
|  | RS09570 | PH domain-containing protein | +3.2029 | <0.001 |
|  | RS13770 | TrmB family transcriptional regulator | +3.1981 | <0.001 |
|  | RS07580 | DUF177 domain-containing protein | +3.1611 | <0.001 |
|  | RS02110 | divalent metal cation transporter | +3.1593 | <0.001 |
|  | RS02865 | YitT family protein | +3.1388 | <0.001 |
|  | RS08015 | putative DNA-binding protein | +3.1272 | <0.001 |
|  | RS15025 | NupC/NupG family nucleoside CNT transporter | +3.1260 | <0.001 |
|  | RS04530 | cold-shock protein | +3.0852 | <0.001 |
|  | RS05650 | thiol management oxidoreductase | +3.0676 | <0.001 |
|  | RS06755 | DUF1836 domain-containing protein | +3.0567 | <0.001 |
|  | RS17115 | ABC transporter | +3.0534 | <0.001 |
|  | RS16650 | protein YvfG | +3.0520 | <0.001 |
|  | RS08130 | flagellar hook-basal body complex protein FliE | +3.0342 | <0.001 |
|  | RS08960 | aspartyl-phosphate phosphatase Spo0E family protein | +3.0180 | <0.001 |
|  | RS15670 | Response regulator receiver domain | +2.9938 | <0.001 |
|  | RS11240 | Na+/H+ antiporter NhaC | +2.9890 | <0.001 |
|  | RS01345 | CDP-diacylglycerol--serine O-phosphatidyltransferase | +2.9832 | <0.001 |
|  | RS09410 | hypothetical protein | +2.9577 | 0.0005 |
|  | RS08100 | tyrosine recombinase XerC | +2.9551 | <0.001 |
|  | RS06740 | RNA polymerase sigma factor SigI | +2.9532 | <0.001 |
|  | RS13775 | AzlC family ABC transporter permease | +2.9471 | <0.001 |
|  | RS18775 | winged helix-turn-helix transcriptional regulator | +2.9338 | 0.0448 |
|  | RS09065 | alanine:cation symporter family protein | +2.9331 | <0.001 |
|  | RS09670 | YojF family protein | +2.9213 | 0.0140 |
|  | RS18725 | branched-chain amino acid aminotransferase | +2.9187 | <0.001 |
|  | RS02070 | hypothetical protein | +2.9029 | <0.001 |
|  | RS03265 | Fur-regulated basic protein FbpA | +2.8947 | <0.001 |
|  | RS07655 | Glycosyltransferase family 28 N-terminal domain | +2.8894 | <0.001 |
|  | RS06835 | aspartyl-phosphate phosphatase Spo0E family protein | +2.8856 | <0.001 |
|  | RS01565 | TerD family protein | +2.8611 | <0.001 |
|  | RS10550 | cell division regulator GpsB | +2.8470 | <0.001 |
|  | RS09335 | site-specific integrase | +2.8454 | <0.001 |
|  | RS03155 | PspA/IM30 family protein | +2.8325 | <0.001 |
|  | RS10955 | genetic competence negative regulator | +2.8221 | <0.001 |
|  | RS17860 | hypothetical protein | +2.7993 | 0.0080 |
|  | RS08770 | MerR family transcriptional regulator | +2.7932 | <0.001 |
|  | RS07010 | hypothetical protein | +2.7880 | <0.001 |
|  | RS09645 | glycosyl transferase family 1 | +2.7655 | <0.001 |
|  | RS04210 | aromatic acid exporter family protein | +2.7431 | <0.001 |
|  | RS17620 | DUF5082 family protein | +2.7431 | <0.001 |
|  | RS15610 | MarR family transcriptional regulator | +2.7409 | 0.0018 |
|  | RS18810 | Phosphomannose isomerase type I | +2.7400 | <0.001 |
|  | RS10210 | MGMT family protein | +2.7312 | <0.001 |
|  | RS11840 | rhodanese-like domain-containing protein | +2.7270 | <0.001 |
|  | RS19100 | hut operon transcriptional regulator HutP | +2.7254 | <0.001 |
|  | RS04965 | MarR family | +2.7206 | <0.001 |
|  | RS14220 | membrane protein insertion efficiency factor YidD | +2.7088 | <0.001 |
|  | RS05610 | GNAT family N-acetyltransferase | +2.7081 | <0.001 |
|  | RS19620 | hypothetical protein | +2.7056 | <0.001 |
|  | RS05340 | MATE family efflux transporter | +2.7035 | <0.001 |
|  | RS13895 | Putative methyltransferase | +2.7000 | <0.001 |
|  | RS04740 | MerR family transcriptional regulator | +2.6949 | <0.001 |
|  | RS03785 | YfmQ family protein | +2.6894 | <0.001 |
|  | RS03220 | GABA permease | +2.6842 | <0.001 |
|  | RS03085 | MoaC family | +2.6697 | <0.001 |
|  | RS03765 | hypothetical protein | +2.6675 | 0.0324 |
|  | RS07360 | aminotransferase class I/II-fold pyridoxal phosphate-dependent enzyme | +2.6662 | <0.001 |
|  | RS02440 | STAS domain-containing protein | +2.6381 | <0.001 |
|  | RS07375 | hypothetical protein | +2.6361 | <0.001 |
|  | RS05825 | esterase family protein | +2.6224 | <0.001 |
|  | RS18870 | ABC transporter | +2.6146 | <0.001 |
|  | RS13750 | type 1 glutamine amidotransferase | +2.6143 | <0.001 |
|  | RS16385 | winged helix-turn-helix transcriptional regulator | +2.5909 | <0.001 |
|  | RS00565 | transcriptional regulator CtsR | +2.5878 | <0.001 |
|  | RS09070 | sugar kinase | +2.5789 | <0.001 |
|  | RS12640 | O-methyltransferase | +2.5766 | <0.001 |
|  | RS08330 | ribosome maturation factor RimP | +2.5629 | <0.001 |
|  | RS12100 | nucleotidase | +2.5595 | <0.001 |
|  | RS11530 | aromatic acid exporter family protein | +2.5516 | <0.001 |
|  | RS17495 | acetolactate synthase AlsS | +2.5368 | <0.001 |
|  | RS19615 | YybS family protein | +2.5298 | <0.001 |
|  | RS11445 | antiterminator LoaP | +2.5294 | <0.001 |
|  | RS11875 | transcriptional regulator SinR | +2.5290 | <0.001 |
|  | RS17545 | Lrp/AsnC family transcriptional regulator | +2.5269 | <0.001 |
|  | RS17475 | ribose ABC transporter substrate-binding protein RbsB | +2.5229 | <0.001 |
|  | RS12085 | LysM peptidoglycan-binding domain-containing protein | +2.5227 | 0.0083 |
|  | RS07535 | YlbF family regulator | +2.5152 | <0.001 |
|  | RS11215 | transcriptional repressor | +2.5145 | <0.001 |
|  | RS17460 | D-ribose pyranase | +2.5131 | <0.001 |
|  | RS06875 | 7-cyano-7-deazaguanine synthase QueC | +2.5098 | <0.001 |
|  | RS17465 | ABC transporter | +2.5056 | <0.001 |
|  | RS02795 | Putative FMN-binding domain | +2.5014 | <0.001 |
|  | RS07175 | DeoR/GlpR transcriptional regulator | +2.4930 | <0.001 |
|  | RS07225 | AbrB/MazE/SpoVT family DNA-binding domain-containing protein | +2.4911 | <0.001 |
|  | RS05520 | ComZ family protein | +2.4894 | 0.0061 |
|  | RS11520 | stressosome-associated protein Prli42 | +2.4830 | <0.001 |
|  | RS17240 | YigZ family protein | +2.4817 | <0.001 |
|  | RS02380 | thioredoxin family protein | +2.4806 | <0.001 |
|  | RS11110 | hypothetical protein | +2.4742 | <0.001 |
|  | RS12125 | DEAD/DEAH box helicase | +2.4721 | <0.001 |
|  | RS08955 | YneF family protein | +2.4710 | <0.001 |
|  | RS12975 | septum formation inhibitor Maf | +2.4506 | <0.001 |
|  | RS13865 | thioredoxin family protein | +2.4484 | <0.001 |
|  | RS10655 | DUF5590 domain-containing protein | +2.4402 | <0.001 |
|  | RS11495 | hypothetical protein | +2.4359 | <0.001 |
|  | RS15560 | Glycine cleavage H-protein | +2.4341 | <0.001 |
|  | RS13265 | cell division protein ZapA | +2.4333 | <0.001 |
|  | RS17940 | ATP synthase subunit I | +2.4313 | <0.001 |
|  | RS02300 | general stress protein | +2.4196 | <0.001 |
|  | RS13155 | glutamate racemase | +2.4128 | <0.001 |
|  | RS13390 | translation initiation factor IF-3 | +2.4094 | <0.001 |
|  | RS02490 | SprT family protein | +2.4078 | <0.001 |
|  | RS08825 | hypothetical protein | +2.4007 | 0.0003 |
|  | RS12110 | YitT family protein | +2.4005 | <0.001 |
|  | RS00680 | class I SAM-dependent methyltransferase | +2.3995 | <0.001 |
|  | RS07815 | phosphoadenylyl-sulfate reductase | +2.3866 | <0.001 |
|  | RS02400 | PH domain-containing protein | +2.3833 | <0.001 |
|  | RS05155 | IDEAL domain-containing protein | +2.3769 | <0.001 |
|  | RS18815 | Glycosyl hydrolase family 26 | +2.3659 | <0.001 |
|  | RS05045 | TetR/AcrR family transcriptional regulator | +2.3565 | <0.001 |
|  | RS09440 | MarR family transcriptional regulator | +2.3503 | 0.0107 |
|  | RS14455 | TrkA family potassium uptake protein | +2.3446 | <0.001 |
|  | RS03180 | Protein of unknown function | +2.3369 | <0.001 |
|  | RS09725 | DUF3311 domain-containing protein | +2.3275 | 0.0124 |
|  | RS10960 | metallophosphoesterase | +2.3258 | <0.001 |
|  | RS17765 | undecaprenyl-diphosphatase | +2.3184 | <0.001 |
|  | RS04770 | glycerophosphodiester phosphodiesterase | +2.3138 | <0.001 |
|  | RS13035 | cytochrome c biogenesis protein | +2.3119 | <0.001 |
|  | RS13715 | GAF domain-containing protein | +2.3022 | <0.001 |
|  | RS15615 | Major Facilitator Superfamily | +2.2962 | <0.001 |
|  | RS02970 | FadR family transcriptional regulator | +2.2932 | <0.001 |
|  | RS03400 | Asp-tRNA(Asn)/Glu-tRNA(Gln) amidotransferase subunit GatC | +2.2925 | <0.001 |
|  | RS12020 | 50S ribosomal protein L33 | +2.2859 | 0.0002 |
|  | RS08445 | GntR family transcriptional regulator | +2.2838 | <0.001 |
|  | RS16435 | GbsR/MarR family transcriptional regulator | +2.2837 | <0.001 |
|  | RS07460 | YlaN family protein | +2.2824 | <0.001 |
|  | RS03160 | TFIIB zinc-binding | +2.2811 | <0.001 |
|  | RS17450 | LacI family DNA-binding transcriptional regulator | +2.2796 | <0.001 |
|  | RS02975 | MFS transporter | +2.2757 | <0.001 |
|  | RS18125 | Nitrate reductase delta subunit | +2.2739 | <0.001 |
|  | RS09045 | CoA-binding protein | +2.2706 | <0.001 |
|  | RS04480 | MarR family transcriptional regulator | +2.2661 | 0.0495 |
|  | RS17990 | UPF0715 family protein | +2.2653 | <0.001 |
|  | RS11000 | phosphoglycerate dehydrogenase | +2.2595 | <0.001 |
|  | RS01485 | TetR/AcrR family transcriptional regulator | +2.2467 | <0.001 |
|  | RS19260 | DeoR/GlpR transcriptional regulator | +2.2299 | <0.001 |
|  | RS10680 | 3-methyl-2-oxobutanoate hydroxymethyltransferase | +2.2273 | <0.001 |
|  | RS18120 | respiratory nitrate reductase subunit gamma | +2.2266 | <0.001 |
|  | RS18025 | NAD-dependent malic enzyme | +2.2249 | <0.001 |
|  | RS12595 | Phosphorylase superfamily | +2.2223 | <0.001 |
|  | RS17245 | LCP family protein | +2.2196 | <0.001 |
|  | RS09730 | sodium:solute symporter | +2.2187 | <0.001 |
|  | RS12005 | Na+/H+ antiporter NhaC | +2.2185 | <0.001 |
|  | RS12540 | TetR/AcrR family transcriptional regulator | +2.2169 | 0.0004 |
|  | RS09930 | hypothetical protein | +2.2065 | <0.001 |
|  | RS09785 | phosphatase PAP2 family protein | +2.2011 | 0.0001 |
|  | RS13825 | DAHP synthetase I family | +2.1947 | <0.001 |
|  | RS06880 | 6-carboxytetrahydropterin synthase QueD | +2.1924 | 0.0002 |
|  | RS10990 | ferredoxin | +2.1917 | <0.001 |
|  | RS07365 | UPF0223 family protein | +2.1901 | <0.001 |
|  | RS12565 | DUF2294 domain-containing protein | +2.1866 | 0.0020 |
|  | RS07660 | UDP-N-acetylmuramate dehydrogenase | +2.1840 | <0.001 |
|  | RS11665 | NADH-dependent flavin oxidoreductase | +2.1835 | <0.001 |
|  | RS05380 | YajQ family cyclic di-GMP-binding protein | +2.1816 | <0.001 |
|  | RS10600 | YppE family protein | +2.1816 | <0.001 |
|  | RS13950 | DeoR/GlpR transcriptional regulator | +2.1669 | <0.001 |
|  | RS18595 | anti-repressor SinI family protein | +2.1607 | <0.001 |
|  | RS17550 | Chromate transporter | +2.1604 | 0.0002 |
|  | RS03875 | DUF1128 domain-containing protein | +2.1583 | 0.0138 |
|  | RS09630 | MoxR family ATPase | +2.1518 | <0.001 |
|  | RS14420 | NfeD family protein | +2.1515 | <0.001 |
|  | RS00670 | 50S ribosomal protein L10 | +2.1495 | <0.001 |
|  | RS18695 | UbiA prenyltransferase family | +2.1454 | <0.001 |
|  | RS02190 | lipid II flippase Amj family protein | +2.1440 | <0.001 |
|  | RS02235 | APC family permease | +2.1384 | <0.001 |
|  | RS02635 | amino acid racemase | +2.1343 | <0.001 |
|  | RS06925 | heavy metal translocating P-type ATPase | +2.1334 | <0.001 |
|  | RS16815 | MFS transporter | +2.1287 | <0.001 |
|  | RS06565 | Amidinotransferase | +2.1212 | <0.001 |
|  | RS07370 | DUF1054 domain-containing protein | +2.1207 | <0.001 |
|  | RS12355 | ComE operon protein 2 | +2.1207 | <0.001 |
|  | RS06885 | 7-carboxy-7-deazaguanine synthase QueE | +2.1166 | <0.001 |
|  | RS02115 | putative hydro-lyase | +2.1106 | <0.001 |
|  | RS13980 | LacI family DNA-binding transcriptional regulator | +2.0990 | <0.001 |
|  | RS14010 | rhodanese-like domain-containing protein | +2.0975 | <0.001 |
|  | RS00210 | cyclic di-AMP receptor DarA | +2.0973 | <0.001 |
|  | RS15205 | MerR family transcriptional regulator | +2.0972 | <0.001 |
|  | RS17965 | low molecular weight protein arginine phosphatase | +2.0875 | <0.001 |
|  | RS02170 | SDR family oxidoreductase | +2.0838 | <0.001 |
|  | RS12025 | hypothetical protein | +2.0792 | <0.001 |
|  | RS17330 | ABC transporter permease | +2.0786 | <0.001 |
|  | RS02765 | hypothetical protein | +2.0771 | 0.0010 |
|  | RS11830 | transcriptional regulator MntR | +2.0754 | <0.001 |
|  | RS15045 | NifU family protein | +2.0717 | <0.001 |
|  | RS07345 | hypothetical protein | +2.0639 | <0.001 |
|  | RS13695 | branched-chain amino acid transport system II carrier protein | +2.0637 | <0.001 |
|  | RS14460 | glucosaminidase domain-containing protein | +2.0616 | <0.001 |
|  | RS03115 | co-chaperone GroES | +2.0527 | <0.001 |
|  | RS09490 | lysozyme family protein | +2.0477 | <0.001 |
|  | RS17345 | glycerol-3-phosphate cytidylyltransferase | +2.0391 | <0.001 |
|  | RS10035 | SGNH/GDSL hydrolase family protein | +2.0368 | <0.001 |
|  | RS10030 | DUF2140 family protein | +2.0343 | <0.001 |
|  | RS08930 | cell division suppressor protein YneA | +2.0267 | 0.0238 |
|  | RS14115 | class I SAM-dependent methyltransferase | +2.0261 | <0.001 |
|  | RS13960 | polysaccharide biosynthesis protein | +2.0248 | <0.001 |
|  | RS19520 | cell wall metabolism DNA-binding response regulator WalR | +2.0246 | <0.001 |
|  | RS07000 | hypothetical protein | +2.0216 | 0.0004 |
|  | RS14805 | YueH family protein | +2.0182 | 0.0018 |
|  | RS12610 | DUF1510 family protein | +2.0182 | <0.001 |
|  | RS06590 | formyltetrahydrofolate deformylase | +2.0162 | <0.001 |
|  | RS07255 | DNA-dependent RNA polymerase auxiliary subunit epsilon family protein | +2.0141 | 0.0003 |
|  | RS18670 | VOC family protein | +2.0124 | <0.001 |
|  | RS13160 | MarR family transcriptional regulator | +2.0122 | <0.001 |
|  | RS10830 | CheR methyltransferase, SAM binding domain | +2.0094 | <0.001 |
|  | RS01675 | hypothetical protein | -11.3040 | <0.001 |
|  | RS03985 | dihydrolipoyl dehydrogenase | -8.3691 | <0.001 |
|  | RS03980 | Biotin-requiring enzyme | -8.2980 | <0.001 |
|  | RS03975 | Transketolase, C-terminal domain | -7.8965 | <0.001 |
|  | RS03970 | Dehydrogenase E1 component | -7.2244 | <0.001 |
|  | RS07680 | S8 family serine peptidase | -7.0812 | <0.001 |
|  | RS15570 | acyl-CoA dehydrogenase family protein | -6.9295 | <0.001 |
|  | RS09155 | non-ribosomal peptide synthetase | -6.7826 | <0.001 |
|  | RS14995 | Bacteriocin class IId cyclical uberolysin-like | -6.4219 | <0.001 |
|  | RS05110 | S8 family serine peptidase | -6.3485 | <0.001 |
|  | RS09175 | short chain dehydrogenase | -6.2301 | <0.001 |
|  | RS11395 | SDR family NAD(P)-dependent oxidoreductase | -6.1285 | <0.001 |
|  | RS08890 | peptidase G2 | -6.0259 | <0.001 |
|  | RS09185 | CoA transferase subunit A | -5.9925 | <0.001 |
|  | RS15575 | acetyl-CoA C-acetyltransferase | -5.9870 | <0.001 |
|  | RS09160 | non-ribosomal peptide synthetase | -5.9227 | <0.001 |
|  | RS09180 | CoA transferase subunit B | -5.9106 | <0.001 |
|  | RS04195 | hypothetical protein | -5.9089 | <0.001 |
|  | RS07505 | cytochrome c oxidase assembly factor CtaG | -5.8931 | <0.001 |
|  | RS08870 | lytic polysaccharide monooxygenase | -5.8423 | <0.001 |
|  | RS11390 | SDR family NAD(P)-dependent oxidoreductase | -5.7588 | <0.001 |
|  | RS07500 | cytochrome c oxidase subunit IVB | -5.6769 | <0.001 |
|  | RS09165 | non-ribosomal peptide synthetase | -5.6738 | <0.001 |
|  | RS11400 | SDR family NAD(P)-dependent oxidoreductase | -5.6150 | <0.001 |
|  | RS07495 | cytochrome (ubi)quinol oxidase subunit III | -5.5882 | <0.001 |
|  | RS15935 | zinc-dependent metalloprotease | -5.5505 | <0.001 |
|  | RS02915 | Methyltransferase domain | -5.4510 | <0.001 |
|  | RS07490 | cytochrome c oxidase subunit I | -5.4474 | <0.001 |
|  | RS11385 | zinc-binding dehydrogenase | -5.4464 | <0.001 |
|  | RS11405 | SDR family NAD(P)-dependent oxidoreductase | -5.3735 | <0.001 |
|  | RS11410 | KR domain-containing protein | -5.2908 | <0.001 |
|  | RS06245 | S9 family peptidase | -5.1361 | <0.001 |
|  | RS06250 | PhrA family phosphatase inhibitor | -5.1220 | <0.001 |
|  | RS09190 | GntP family permease | -5.1065 | <0.001 |
|  | RS10215 | hypothetical protein | -5.0673 | <0.001 |
|  | RS02910 | NUDIX hydrolase | -5.0636 | <0.001 |
|  | RS11380 | cytochrome P450 | -5.0568 | <0.001 |
|  | RS06255 | N-acetylmuramoyl-L-alanine amidase | -4.9930 | <0.001 |
|  | RS09755 | Methyltransferase domain | -4.9330 | <0.001 |
|  | RS04900 | hypothetical protein | -4.8871 | <0.001 |
|  | RS18520 | S8 family serine peptidase | -4.8610 | <0.001 |
|  | RS10740 | menaquinol-cytochrome c reductase cytochrome b/c subunit | -4.8564 | <0.001 |
|  | RS08885 | hypothetical protein | -4.8164 | <0.001 |
|  | RS11415 | SDR family NAD(P)-dependent oxidoreductase | -4.7907 | <0.001 |
|  | RS08640 | amino acid adenylation domain-containing protein | -4.7901 | <0.001 |
|  | RS08645 | polyketide synthase dehydratase domain-containing protein | -4.7475 | <0.001 |
|  | RS07485 | cytochrome c oxidase subunit II | -4.7213 | <0.001 |
|  | RS08630 | SDR family NAD(P)-dependent oxidoreductase | -4.7212 | <0.001 |
|  | RS11375 | hydroxymethylglutaryl-CoA synthase family protein | -4.6745 | <0.001 |
|  | RS11370 | enoyl-CoA hydratase/isomerase family protein | -4.5743 | <0.001 |
|  | RS08635 | SDR family NAD(P)-dependent oxidoreductase | -4.5636 | <0.001 |
|  | RS02625 | sulfurtransferase TusA family protein | -4.4766 | 0.0297 |
|  | RS19460 | cupin-like domain-containing protein | -4.4695 | <0.001 |
|  | RS07300 | SDR family NAD(P)-dependent oxidoreductase | -4.4613 | <0.001 |
|  | RS06535 | peptide ABC transporter substrate-binding protein | -4.3546 | <0.001 |
|  | RS01275 | sigma-G-dependent sporulation-specific acid-soluble spore protein CsgA | -4.3390 | <0.001 |
|  | RS15905 | hypothetical protein | -4.3093 | <0.001 |
|  | RS07235 | gamma-glutamylcyclotransferase | -4.3080 | <0.001 |
|  | RS07630 | stage V sporulation protein D | -4.3072 | <0.001 |
|  | RS08820 | hypothetical protein | -4.2812 | <0.001 |
|  | RS13215 | Electron transfer flavoprotein domain | -4.2400 | <0.001 |
|  | RS15830 | Periplasmic binding protein | -4.2355 | <0.001 |
|  | RS16345 | transfer-messenger RNA | -4.2339 | <0.001 |
|  | RS01820 | Thioesterase domain | -4.2228 | <0.001 |
|  | RS10545 | RNase P RNA component class B | -4.2135 | <0.001 |
|  | RS11175 | RNA polymerase sporulation sigma factor SigF | -4.1979 | <0.001 |
|  | RS06545 | dipeptide epimerase | -4.1536 | <0.001 |
|  | RS01630 | starch-binding protein | -4.1517 | <0.001 |
|  | RS11360 | MBL fold metallo-hydrolase | -4.1388 | <0.001 |
|  | RS06550 | C40 family peptidase | -4.1189 | <0.001 |
|  | RS03620 | methyl-accepting chemotaxis protein | -4.1021 | <0.001 |
|  | RS09735 | glucosylceramidase | -4.0938 | <0.001 |
|  | RS07310 | SDR family NAD(P)-dependent oxidoreductase | -4.0595 | <0.001 |
|  | RS02270 | Nramp family divalent metal transporter | -4.0499 | <0.001 |
|  | RS10230 | hypothetical protein | -4.0445 | <0.001 |
|  | RS09530 | 6S RNA | -4.0390 | <0.001 |
|  | RS07285 | SDR family NAD(P)-dependent oxidoreductase | -4.0102 | <0.001 |
|  | RS06575 | GNAT family N-acetyltransferase | -3.9945 | <0.001 |
|  | RS03615 | aldehyde dehydrogenase family protein | -3.9897 | <0.001 |
|  | RS19440 | arginase | -3.9743 | <0.001 |
|  | RS01015 | Zinc-binding dehydrogenase | -3.9713 | <0.001 |
|  | RS19430 | GNAT family N-acetyltransferase | -3.9674 | <0.001 |
|  | RS01815 | surfactin non-ribosomal peptide synthetase SrfAC | -3.9671 | <0.001 |
|  | RS19445 | amino acid permease | -3.9373 | <0.001 |
|  | RS15820 | iron ABC transporter permease | -3.9243 | <0.001 |
|  | RS06970 | methyl-accepting chemotaxis protein | -3.9060 | <0.001 |
|  | RS12745 | 6S RNA | -3.8840 | <0.001 |
|  | RS15640 | ABC transporter | -3.8780 | <0.001 |
|  | RS09870 | hypothetical protein | -3.8653 | 0.0003 |
|  | RS15580 | 3-hydroxyacyl-CoA dehydrogenase/enoyl-CoA hydratase family protein | -3.8498 | <0.001 |
|  | RS06790 | methylated-DNA--[protein]-cysteine S-methyltransferase | -3.8490 | <0.001 |
|  | RS11450 | LysR family transcriptional regulator | -3.8013 | <0.001 |
|  | RS04145 | SH3 domain-containing protein | -3.7923 | <0.001 |
|  | RS17805 | Glycosyl hydrolases family 11 | -3.7673 | <0.001 |
|  | RS11180 | anti-sigma F factor | -3.7662 | <0.001 |
|  | RS11420 | SDR family oxidoreductase | -3.7361 | <0.001 |
|  | RS19450 | Aminotransferase class-III | -3.7297 | <0.001 |
|  | RS14595 | hydrolase | -3.7261 | <0.001 |
|  | RS18085 | (Fe-S)-binding protein | -3.7008 | <0.001 |
|  | RS01470 | pyroglutamyl-peptidase I | -3.6848 | <0.001 |
|  | RS15635 | YusU family protein | -3.6646 | <0.001 |
|  | RS15155 | Aminotransferase class-V | -3.6597 | <0.001 |
|  | RS11425 | long-chain fatty acid--CoA ligase | -3.6442 | <0.001 |
|  | RS01270 | DUF4879 domain-containing protein | -3.6369 | <0.001 |
|  | RS07290 | SDR family NAD(P)-dependent oxidoreductase | -3.6234 | <0.001 |
|  | RS00910 | DinB family protein | -3.6174 | <0.001 |
|  | RS04015 | ABC transporter ATP-binding protein | -3.6134 | <0.001 |
|  | RS07305 | SDR family NAD(P)-dependent oxidoreductase | -3.5972 | <0.001 |
|  | RS05400 | hypothetical protein | -3.5575 | 0.0001 |
|  | RS05095 | fatty acid--CoA ligase family protein | -3.5475 | <0.001 |
|  | RS02000 | Periplasmic binding protein | -3.5458 | <0.001 |
|  | RS06225 | class II lanthipeptide%2C LchA2/BrtA2 family | -3.5437 | 0.0001 |
|  | RS09310 | D-alanyl-D-alanine carboxypeptidase/D-alanyl-D-alanine-endopeptidase | -3.5408 | <0.001 |
|  | RS01805 | surfactin non-ribosomal peptide synthetase SrfAA | -3.5373 | <0.001 |
|  | RS10745 | cytochrome b6 | -3.5369 | <0.001 |
|  | RS01505 | aldo/keto reductase | -3.5344 | <0.001 |
|  | RS14540 | YjbQ family protein | -3.5301 | <0.001 |
|  | RS00095 | glycerate kinase | -3.5051 | <0.001 |
|  | RS12550 | hypothetical protein | -3.5019 | <0.001 |
|  | RS16440 | glycine betaine/carnitine/choline/choline sulfate ABC transporter permease OpuCD | -3.4829 | <0.001 |
|  | RS01810 | surfactin non-ribosomal peptide synthetase SrfAB | -3.4683 | <0.001 |
|  | RS15225 | FAD-binding oxidoreductase | -3.4611 | <0.001 |
|  | RS11975 | DUF2759 domain-containing protein | -3.4491 | <0.001 |
|  | RS07295 | SDR family NAD(P)-dependent oxidoreductase | -3.4351 | <0.001 |
|  | RS08625 | non-ribosomal peptide synthetase 1 | -3.4324 | <0.001 |
|  | RS14530 | hypothetical protein | -3.4264 | <0.001 |
|  | RS06070 | YjfB family protein | -3.4217 | <0.001 |
|  | RS06555 | ATP-binding cassette domain-containing protein | -3.4105 | <0.001 |
|  | RS18375 | Aldehyde dehydrogenase family | -3.4006 | <0.001 |
|  | RS04895 | enoyl-CoA hydratase | -3.3834 | <0.001 |
|  | RS09000 | aconitate hydratase AcnA | -3.3744 | <0.001 |
|  | RS07780 | dihydroorotase | -3.3613 | <0.001 |
|  | RS04050 | acyltransferase family protein | -3.3535 | <0.001 |
|  | RS07330 | pyruvate dehydrogenase complex E1 component subunit beta | -3.3236 | <0.001 |
|  | RS11430 | acyl carrier protein | -3.3119 | <0.001 |
|  | RS15050 | S9 family peptidase | -3.3090 | <0.001 |
|  | RS19115 | imidazolonepropionase | -3.3059 | <0.001 |
|  | RS14910 | alpha/beta hydrolase | -3.3051 | <0.001 |
|  | RS02195 | DUF3817 domain-containing protein | -3.3010 | <0.001 |
|  | RS19420 | hypothetical protein | -3.2643 | <0.001 |
|  | RS05175 | hypothetical protein | -3.2363 | <0.001 |
|  | RS06220 | class II lanthipeptide%2C LchA2/BrtA2 family | -3.2287 | <0.001 |
|  | RS19415 | hypothetical protein | -3.2286 | <0.001 |
|  | RS10510 | purine permease | -3.2261 | <0.001 |
|  | RS03680 | pectate lyase | -3.2220 | <0.001 |
|  | RS17155 | Flagellar protein FliS | -3.2134 | <0.001 |
|  | RS14205 | YtzI protein | -3.2098 | <0.001 |
|  | RS11435 | D-fructose-6-phosphate amidotransferase | -3.1984 | <0.001 |
|  | RS17150 | flagella biosynthesis regulatory protein FliT | -3.1984 | <0.001 |
|  | RS05405 | DegV family protein & | -3.1852 | <0.001 |
|  | RS05600 | oligopeptide ABC transporter ATP-binding protein OppF | -3.1821 | <0.001 |
|  | RS19120 | formimidoylglutamase | -3.1791 | <0.001 |
|  | RS19465 | ABC-F family ATP-binding cassette domain-containing protein | -3.1705 | <0.001 |
|  | RS11440 | ACP S-malonyltransferase | -3.1480 | <0.001 |
|  | RS07320 | serine hydrolase | -3.1411 | <0.001 |
|  | RS15150 | tetracycline resistance MFS efflux pump | -3.1239 | <0.001 |
|  | RS19050 | MerR family transcriptional regulator | -3.1165 | <0.001 |
|  | RS18015 | chromosome-anchoring protein RacA | -3.0892 | <0.001 |
|  | RS14535 | nitronate monooxygenase | -3.0797 | <0.001 |
|  | RS19125 | amino acid permease | -3.0686 | <0.001 |
|  | RS01005 | Aminotransferase class-III | -3.0630 | <0.001 |
|  | RS17165 | flagellin | -3.0621 | <0.001 |
|  | RS01990 | FecCD transport family | -3.0402 | <0.001 |
|  | RS17145 | hypothetical protein | -3.0348 | <0.001 |
|  | RS17160 | flagellar hook-associated protein 2 | -3.0342 | <0.001 |
|  | RS09150 | carbohydrate-binding protein | -3.0205 | <0.001 |
|  | RS07315 | Polyketide synthase dehydratase | -3.0193 | <0.001 |
|  | RS01050 | AraC family transcriptional regulator & | -3.0117 | <0.001 |
|  | RS06530 | ABC transporter ATP-binding protein | -3.0046 | <0.001 |
|  | RS02055 | DUF1775 domain-containing protein | -2.9914 | <0.001 |
|  | RS13220 | Electron transfer flavoprotein domain | -2.9848 | <0.001 |
|  | RS09280 | DUF1360 domain-containing protein | -2.9673 | <0.001 |
|  | RS13455 | glyceraldehyde-3-phosphate dehydrogenase | -2.9552 | <0.001 |
|  | RS13225 | enoyl-CoA hydratase | -2.9541 | <0.001 |
|  | RS11035 | AhpC/TSA family | -2.9365 | <0.001 |
|  | RS14590 | NAD(P)H-dependent oxidoreductase | -2.9349 | <0.001 |
|  | RS01030 | alpha/beta hydrolase | -2.9293 | <0.001 |
|  | RS19295 | fatty acid desaturase | -2.9196 | <0.001 |
|  | RS14515 | HAMP domain-containing protein | -2.9147 | <0.001 |
|  | RS08180 | flagellar FlbD family protein | -2.8789 | <0.001 |
|  | RS09640 | peptidoglycan endopeptidase | -2.8646 | <0.001 |
|  | RS19110 | urocanate hydratase | -2.8631 | <0.001 |
|  | RS19470 | MFS transporter | -2.8597 | <0.001 |
|  | RS18370 | M20/M25/M40 family metallo-hydrolase | -2.8591 | <0.001 |
|  | RS01055 | DUF1343 domain-containing protein | -2.8584 | <0.001 |
|  | RS01045 | ABC transporter substrate-binding protein | -2.8517 | <0.001 |
|  | RS06205 | plantaricin C family lantibiotic | -2.8333 | 0.0202 |
|  | RS08250 | chemotaxis protein CheA | -2.8325 | <0.001 |
|  | RS08265 | chemotaxis protein CheD | -2.8139 | <0.001 |
|  | RS17065 | CsbA family protein | -2.8034 | <0.001 |
|  | RS04230 | YgzB family protein | -2.7895 | <0.001 |
|  | RS14510 | methyl-accepting chemotaxis protein | -2.7891 | <0.001 |
|  | RS11355 | SDR family oxidoreductase | -2.7791 | <0.001 |
|  | RS01000 | MFS transporter | -2.7429 | <0.001 |
|  | RS13745 | hypothetical protein | -2.7320 | <0.001 |
|  | RS00310 | septation regulator SpoVG | -2.7269 | <0.001 |
|  | RS19435 | aspartate phosphatase | -2.7187 | <0.001 |
|  | RS06785 | PAS domain-containing sensor histidine kinase | -2.7098 | <0.001 |
|  | RS17410 | C40 family peptidase | -2.7003 | <0.001 |
|  | RS09355 | Helix-turn-helix domain | -2.6977 | <0.001 |
|  | RS15745 | MFS transporter | -2.6946 | <0.001 |
|  | RS11505 | Peptidase dimerisation domain | -2.6931 | <0.001 |
|  | RS08260 | chemotaxis protein CheC | -2.6896 | <0.001 |
|  | RS19475 | ABC transporter substrate-binding protein | -2.6840 | <0.001 |
|  | RS09760 | hypothetical protein | -2.6837 | <0.001 |
|  | RS08600 | ACP S-malonyltransferase | -2.6653 | <0.001 |
|  | RS08240 | MinD/ParA family protein | -2.6620 | <0.001 |
|  | RS06525 | ABC transporter permease | -2.6372 | <0.001 |
|  | RS09285 | non-ribosomal peptide synthase | -2.6359 | <0.001 |
|  | RS09145 | glucuronoxylanase xynC | -2.6216 | <0.001 |
|  | RS01970 | PhrC/PhrF family phosphatase-inhibitory pheromone | -2.6191 | 0.0399 |
|  | RS04045 | Flavodoxin-like fold | -2.6171 | <0.001 |
|  | RS01165 | aldo/keto reductase | -2.6131 | <0.001 |
|  | RS09005 | AhpC/TSA family | -2.6053 | <0.001 |
|  | RS09635 | superoxide dismutase family protein | -2.5969 | <0.001 |
|  | RS15240 | chitosanase | -2.5913 | <0.001 |
|  | RS07785 | glutamine-hydrolyzing carbamoyl-phosphate synthase small subunit | -2.5905 | <0.001 |
|  | RS19275 | molecular chaperone HtpG | -2.5837 | <0.001 |
|  | RS09585 | aldehyde dehydrogenase family protein | -2.5803 | <0.001 |
|  | RS01010 | hypothetical protein | -2.5733 | <0.001 |
|  | RS02060 | copper resistance protein CopC | -2.5643 | <0.001 |
|  | RS18380 | Glu/Leu/Phe/Val dehydrogenase | -2.5621 | <0.001 |
|  | RS05415 | proteinase inhibitor | -2.5549 | <0.001 |
|  | RS12865 | YhcN/YlaJ family sporulation lipoprotein | -2.5417 | <0.001 |
|  | RS08175 | flagellar basal body rod protein FlgG | -2.5411 | <0.001 |
|  | RS07790 | carbamoyl-phosphate synthase large subunit | -2.5352 | <0.001 |
|  | RS07005 | chemotaxis protein CheV | -2.5327 | <0.001 |
|  | RS19425 | aldo/keto reductase | -2.5302 | <0.001 |
|  | RS10750 | Cytochrome B6-F complex Fe-S subunit | -2.5253 | <0.001 |
|  | RS07340 | dihydrolipoyl dehydrogenase | -2.5195 | <0.001 |
|  | RS14160 | phosphoenolpyruvate carboxykinase (ATP) | -2.5030 | <0.001 |
|  | RS05055 | ketoacyl-ACP synthase III | -2.5012 | <0.001 |
|  | RS04775 | YhdX family protein | -2.4937 | <0.001 |
|  | RS04055 | phosphoenolpyruvate synthase | -2.4805 | <0.001 |
|  | RS05595 | ABC transporter ATP-binding protein | -2.4788 | <0.001 |
|  | RS05140 | globin-coupled sensor protein | -2.4713 | <0.001 |
|  | RS12810 | stage V sporulation protein B | -2.4691 | <0.001 |
|  | RS11490 | DNA polymerase IV | -2.4624 | <0.001 |
|  | RS04035 | protoheme IX farnesyltransferase | -2.4575 | <0.001 |
|  | RS01035 | iron ABC transporter permease | -2.4495 | <0.001 |
|  | RS18285 | DUF1700 domain-containing protein | -2.4451 | <0.001 |
|  | RS08195 | flagellar motor switch phosphatase FliY | -2.4280 | <0.001 |
|  | RS03095 | twin-arginine translocase TatA/TatE family subunit | -2.4278 | <0.001 |
|  | RS04910 | ABC transporter permease | -2.4260 | <0.001 |
|  | RS02550 | BCCT family transporter | -2.3992 | <0.001 |
|  | RS06540 | LD-carboxypeptidase | -2.3933 | <0.001 |
|  | RS14945 | YuiB family protein | -2.3931 | <0.001 |
|  | RS06010 | Glycosyl hydrolase family 53 | -2.3712 | <0.001 |
|  | RS17775 | M23 family metallopeptidase | -2.3668 | <0.001 |
|  | RS14845 | type VII secretion protein EssC | -2.3611 | <0.001 |
|  | RS18680 | S8 family serine peptidase | -2.3457 | <0.001 |
|  | RS18730 | 6-phospho-beta-glucosidase | -2.3445 | <0.001 |
|  | RS00580 | ATP-dependent Clp protease ATP-binding subunit | -2.3426 | <0.001 |
|  | RS11185 | anti-sigma F factor antagonist | -2.3300 | <0.001 |
|  | RS15070 | spore coat protein YutH | -2.3274 | <0.001 |
|  | RS08965 | cytochrome c biogenesis protein CcdA | -2.3262 | <0.001 |
|  | RS03330 | MGS-like domain | -2.3231 | <0.001 |
|  | RS06655 | S8 family peptidase | -2.3217 | <0.001 |
|  | RS06520 | ABC transporter permease | -2.3212 | <0.001 |
|  | RS03385 | phosphotransferase enzyme family protein | -2.3179 | <0.001 |
|  | RS03335 | phosphoribosylamine--glycine ligase | -2.3159 | <0.001 |
|  | RS02275 | hypothetical protein | -2.3131 | <0.001 |
|  | RS08200 | chemotaxis protein CheY | -2.3033 | <0.001 |
|  | RS07770 | uracil transporter | -2.2965 | <0.001 |
|  | RS09315 | aldose 1-epimerase | -2.2821 | <0.001 |
|  | RS08535 | glycine C-acetyltransferase | -2.2787 | <0.001 |
|  | RS03845 | Calcineurin-like phosphoesterase | -2.2771 | <0.001 |
|  | RS07775 | aspartate carbamoyltransferase catalytic subunit | -2.2752 | <0.001 |
|  | RS16445 | osmoprotectant ABC transporter substrate-binding protein | -2.2708 | <0.001 |
|  | RS03315 | amidophosphoribosyltransferase | -2.2702 | <0.001 |
|  | RS09885 | 3-phytase | -2.2695 | <0.001 |
|  | RS05310 | fumarylacetoacetate hydrolase family protein | -2.2649 | <0.001 |
|  | RS01920 | amino acid ABC transporter substrate-binding protein | -2.2630 | 0.0001 |
|  | RS18935 | catalase | -2.2583 | <0.001 |
|  | RS14875 | YukJ family protein | -2.2489 | <0.001 |
|  | RS03325 | Formyl transferase | -2.2457 | <0.001 |
|  | RS16455 | ABC transporter | -2.2406 | <0.001 |
|  | RS08525 | stage V sporulation protein SpoVS | -2.2362 | <0.001 |
|  | RS17900 | F0F1 ATP synthase subunit epsilon | -2.2354 | <0.001 |
|  | RS11350 | D-serine ammonia-lyase | -2.2325 | <0.001 |
|  | RS13830 | bacillithiol system redox-active protein YtxJ | -2.2244 | <0.001 |
|  | RS07795 | dihydroorotate dehydrogenase electron transfer subunit | -2.2150 | <0.001 |
|  | RS16630 | PLP-dependent aminotransferase family protein | -2.2117 | <0.001 |
|  | RS14705 | guanosine ABC transporter permease NupQ & | -2.2089 | <0.001 |
|  | RS09375 | glutamate synthase small subunit | -2.2020 | <0.001 |
|  | RS00460 | dihydropteroate synthase | -2.1886 | <0.001 |
|  | RS14670 | thiol-disulfide oxidoreductase DCC family protein | -2.1818 | <0.001 |
|  | RS16705 | glycosyltransferase family 2 protein | -2.1810 | 0.0001 |
|  | RS07990 | acyl carrier protein | -2.1711 | <0.001 |
|  | RS07985 | short chain dehydrogenase | -2.1593 | <0.001 |
|  | RS11605 | methylisocitrate lyase | -2.1568 | <0.001 |
|  | RS10515 | Phosphoribosyl transferase domain | -2.1409 | 0.0001 |
|  | RS09290 | amino acid adenylation domain-containing protein | -2.1408 | <0.001 |
|  | RS04065 | DinB superfamily | -2.1381 | <0.001 |
|  | RS06990 | PAS domain-containing protein | -2.1372 | <0.001 |
|  | RS07335 | pyruvate dehydrogenase complex dihydrolipoyllysine-residue acetyltransferase | -2.1161 | <0.001 |
|  | RS05580 | peptide ABC transporter substrate-binding protein | -2.1113 | <0.001 |
|  | RS07280 | ACP S-malonyltransferase | -2.1111 | <0.001 |
|  | RS03640 | MerR family transcriptional regulator | -2.1079 | <0.001 |
|  | RS01730 | NAD(P)/FAD-dependent oxidoreductase | -2.1066 | <0.001 |
|  | RS13230 | TetR/AcrR family transcriptional regulator | -2.0966 | <0.001 |
|  | RS01715 | Aldehyde dehydrogenase family | -2.0966 | <0.001 |
|  | RS17440 | endospore germination permease | -2.0963 | <0.001 |
|  | RS17905 | F0F1 ATP synthase subunit beta | -2.0885 | <0.001 |
|  | RS12475 | DinB family protein | -2.0861 | <0.001 |
|  | RS15815 | ABC transporter ATP-binding protein | -2.0859 | <0.001 |
|  | RS17705 | flagellar hook-basal body protein | -2.0812 | <0.001 |
|  | RS08255 | chemotaxis protein CheW | -2.0806 | <0.001 |
|  | RS01060 | glycoside hydrolase family 3 C-terminal domain-containing protein | -2.0742 | <0.001 |
|  | RS19180 | Periplasmic binding protein | -2.0583 | 0.0001 |
|  | RS08495 | CDP-alcohol phosphatidyltransferase | -2.0463 | <0.001 |
|  | RS16565 | sugar porter family MFS transporter | -2.0369 | <0.001 |
|  | RS07325 | Dehydrogenase E1 component | -2.0344 | <0.001 |
|  | RS18630 | thiamine phosphate synthase | -2.0310 | <0.001 |
|  | RS03320 | phosphoribosylformylglycinamidine cyclo-ligase | -2.0294 | <0.001 |
|  | RS00350 | polysaccharide biosynthesis protein | -2.0267 | <0.001 |
|  | RS16665 | acetyltransferase | -2.0217 | 0.0489 |
|  | RS04810 | universal stress protein & | -2.0210 | <0.001 |
|  | RS03310 | phosphoribosylformylglycinamidine synthase subunit PurL | -2.0187 | <0.001 |
|  | RS05410 | YitT family protein | -2.0146 | <0.001 |
|  | RS11335 | DUF4083 family protein | -2.0139 | <0.001 |
|  | RS01305 | DUF2651 family protein | -2.0106 | 0.0012 |
|  | RS17140 | ribosome-associated translation inhibitor RaiA | -2.0087 | <0.001 |
|  | RS05565 | ABC transporter permease | -2.0017 | 0.0090 |

**Table S4** The Gene Ontology (GO) functional annotation of differentially expressed genes Se-enriched *B. amyloliquefaciens* C-1 (BP, Biological Process; CC, cellular Component; MF, Molecular Function).

| Stage | Category | GO ID | Description | *P_adj_* | Gene Count | Regulation |
| --- | --- | --- | --- | --- | --- | --- |
| Vegetative stage | CC | 0032991 | protein-containing complex | 0.0058 | 11 | Down |
|  | MF | 0004553 | hydrolase activity, hydrolyzing O-glycosyl compounds | 0.0041 | 11 | Down |
|  | MF | 0016798 | hydrolase activity, acting on glycosyl bonds | 0.0048 | 11 | Down |
| Spores stage | BP | 0006935 | chemotaxis | 0.0095 | 9 | Down |
|  | BP | 0042330 | taxis | 0.0095 | 9 | Down |
|  | BP | 0040011 | locomotion | 0.0206 | 14 | Down |
|  | BP | 0006139 | nucleobase-containig compound metabolic process | 0.0043 | 112 | Up |
|  | BP | 0090304 | nucleic acid metabolic process | 0.0043 | 95 | Up |
|  | BP | 0006725 | cellular aromatic compound metabolic process | 0.0095 | 119 | Up |
|  | BP | 0046483 | heterocycle metabolic process | 0.0098 | 120 | Up |
|  | BP | 0034641 | cellular nitrogen compound metabolic process | 0.0098 | 128 | Up |
|  | BP | 1901360 | organic cyclic compound metabolic process | 0.0098 | 120 | Up |
|  | BP | 0016070 | RNA metabolic process | 0.0136 | 76 | Up |
|  | BP | 0010467 | gene expression | 0.0178 | 81 | Up |
|  | BP | 0044249 | cellular biosynthetic process | 0.0178 | 111 | Up |
|  | BP | 0044260 | cellular macromolecule metabolic process | 0.0279 | 92 | Up |
|  | BP | 1901576 | organic substance biosynthetic process | 0.0304 | 111 | Up |
|  | BP | 0034660 | ncRNA metabolic process | 0.0308 | 18 | Up |
|  | BP | 0071840 | cellular component organization or biogenesis | 0.0308 | 21 | Up |
|  | BP | 0034470 | ncRNA processing | 0.0308 | 11 | Up |
|  | BP | 0034645 | cellular macromolecule biosynthetic process | 0.0331 | 76 | Up |
|  | BP | 0009058 | biosynthetic process | 0.0344 | 122 | Up |
|  | BP | 0009059 | macromolecule biosynthetic process | 0.0394 | 76 | Up |
|  | BP | 0043170 | macromolecule metabolic process | 0.0394 | 114 | Up |
|  | BP | 0080090 | regulation of primary metabolic process | 0.0394 | 56 | Up |
|  | BP | 0044271 | cellular nitrogen compound biosynthetic process | 0.0394 | 88 | Up |
|  | BP | 0034654 | nucleobase-containing compound biosynthetic process | 0.0394 | 65 | Up |
|  | BP | 0019222 | regulation of metabolic process | 0.0394 | 56 | Up |
|  | BP | 0009889 | regulation of biosynthetic process | 0.0394 | 55 | Up |
|  | BP | 0010556 | regulation of macromolecule biosynthetic process | 0.0394 | 55 | Up |
|  | BP | 0031326 | regulation of cellular biosynthetic process | 0.0394 | 55 | Up |
|  | BP | 2000112 | regulation of cellular macromolecule biosynthetic process | 0.0394 | 55 | Up |
|  | BP | 0019219 | regulation of nucleobase-containing compound metabolic process | 0.0410 | 55 | Up |
|  | BP | 0031323 | regulation of cellular metabolic process | 0.0410 | 55 | Up |
|  | BP | 0051171 | regulation of nitrogen compound metabolic process | 0.0410 | 55 | Up |
|  | BP | 0006811 | ion transport | 0.0410 | 24 | Up |
|  | BP | 0060255 | regulation of macromolecule metabolic process | 0.0410 | 55 | Up |
|  | BP | 0006355 | regulation of transcription, DNA-templated | 0.0410 | 54 | Up |
|  | BP | 0051252 | regulation of RNA metabolic process | 0.0410 | 54 | Up |
|  | BP | 1903506 | regulation of nucleic acid-templated transcription | 0.0410 | 54 | Up |
|  | BP | 2001141 | regulation of RNA biosynthetic process | 0.0410 | 54 | Up |
| Spores stage | BP | 0008033 | tRNA processing | 0.0410 | 7 | Up |
|  | BP | 0006399 | tRNA metabolic process | 0.0410 | 14 | Up |
|  | BP | 0010468 | regulation of gene expression | 0.0452 | 54 | Up |
|  | BP | 0044085 | cellular component biogenesis | 0.0492 | 11 | Up |

**Table S5 Differentially expressed genes by KEGG pathway categories.**

| Stage | KEGG ID | Description | *P_adj_* | Gene  count | Regulation |
| --- | --- | --- | --- | --- | --- |
| Vegetative stage | bao00500 | Starch and sucrose metabolism | 0.0001 | 7 | Up |
|  | bao02060 | Phosphotransferase system (PTS) | 0.0146 | 4 | Up |
|  | bao00230 | Purine metabolism | 0.0251 | 15 | Down |
|  | bao00190 | Oxidative phosphorylation | 0.0251 | 12 | Down |
| Spores stage | bao00564 | Glycerophospholipid metabolism | 0.0155 | 12 | Up |
|  | bao00051 | Fructose and mannose metabolism | 0.0410 | 11 | Up |
|  | bao00020 | Citrate cycle (TCA cycle) | 0.0003 | 17 | Down |
|  | bao02030 | Bacterial chemotaxis | 0.0025 | 15 | Down |
|  | bao00010 | Glycolysis / Gluconeogenesis | 0.0458 | 19 | Down |
|  | bao00630 | Glyoxylate and dicarboxylate metabolism | 0.0458 | 17 | Down |
|  | bao00190 | Oxidative phosphorylation | 0.0484 | 17 | Down |
|  | bao01200 | Carbon metabolism | 0.0484 | 36 | Down |
